# Mechanism mediating the biosynthesis of the anti-insect volatile (*Z*)-3-hexenyl acetate in *Acacia confusa* Merr., an intercropping plant in tea plantations

**DOI:** 10.1186/s43897-025-00165-z

**Published:** 2025-08-07

**Authors:** Guotai Jian, Jianlong Li, Yong Wu, Chengshun Liu, Ronghua Li, Jiajia Qian, Yongxia Jia, Hanxiang Li, Jinchi Tang, Lanting Zeng

**Affiliations:** 1 Guangdong Provincial Key Laboratory of Applied Botany & State Key Laboratory of Plant Diversity and Specialty Crops, South China Botanical Garden, Chinese Academy of Sciences, No. 723 Xingke Road, Tianhe District, Guangzhou, 510650 China; 2 Tea Research Institute, Guangdong Academy of Agricultural Sciences & Guangdong Provincial Key Laboratory of Tea Plant Resources Innovation and Utilization, No. 6 Dafeng Road, Tianhe District, Guangzhou, 510640 China; 3 Key Laboratory of National Forestry and Grassland Administration on Plant Conservation and Utilization in Southern China, South China Botanical Garden, Chinese Academy of Sciences, No. 723 Xingke Road, Tianhe District, Guangzhou, 510650 China; 4 University of Chinese Academy of Sciences, No. 19A Yuquan Road, Beijing, 100049 China; 5 Yingde City Meteorological Bureau, No. 44 East Education Road, Yingde, Qingyuan 513000 China

**Keywords:** *Camellia sinensis*, *Acacia confuse* Merr., (*Z*)-3-hexenyl acetate, Anti-insect, Biosynthesis

## Abstract

Intercropping tea plants with *Acacia confusa* Merr. offers an environmentally sustainable approach to insect population control in tea plantations. However, the primary compounds in *A. confusa* responsible for this effect and their biosynthetic mechanisms remain undetermined. This study identified (*Z*)-3-hexenyl acetate, (*Z*)-3-hexenol, and 1-hexanol as the major volatiles in *A. confusa*. Field experiments demonstrated that all three compounds affected the tea leafhopper, a significant pest. (*Z*)-3-Hexenyl acetate repelled leafhoppers, while the other two compounds attracted them. Leafhopper feeding on tea leaves significantly decreased after fumigation with (*Z*)-3-hexenyl acetate, potentially altering the metabolism of defensive substances in tea leaves. These findings suggest (*Z*)-3-hexenyl acetate as a crucial component for pest control in tea plantations intercropped with *A. confusa*. Furthermore, the study identified the nucleus- and cytoplasm-localized AcAAT4 in *A. confusa* as responsible for (*Z*)-3-hexenyl acetate biosynthesis. Notably, *AcAAT4* expression and the production of the upstream transcription factor AcMYC2b corresponded to the (*Z*)-3-hexenyl acetate emission pattern. The research also elucidated the positive regulatory effects of nucleus-localized AcMYC2b on *AcAAT4* expression. These findings elucidate the molecular basis of (*Z*)-3-hexenyl acetate emission from *A. confusa* and provide a theoretical foundation for enhancing intercropping practices in tea plantations.

**Supplementary Information:**

The online version contains supplementary material available at 10.1186/s43897-025-00165-z.

## Core

For the first time, the central role of the volatile compound (*Z*)- -hexenyl acetate from *Acacia confusa* in the insect resistance defense of tea plants has been clarified, and its biosynthesis and regulatory mechanisms have been elucidated.

## Gene & accession numbers

All transcriptome files generated for this study were deposited in NGDC (https://ngdc.cncb.accn/gsa/) under Bio Project ID PRJCA036017.

## Introduction

Tea (*Camellia sinensis*) is a significant perennial economic crop that flourishes in warm, humid environments with diffused light (Wang et al. 2018). To optimize tea yields, monoculture practices have been widely adopted, particularly in densely planted strip tea plantations. However, this large-scale monoculture model has negatively impacted tea plant growth conditions. Environmental changes significantly affect light intensity, moisture, and temperature in tea plantations, resulting in diminished natural regulation. Consequently, plants struggle to withstand natural disasters, leading to decreased biodiversity and soil degradation, which ultimately affects tea plantation productivity (Yao et al. 2016). Due to the growing recognition of intercropping benefits, the area dedicated to intercropping in tea plantations has been expanding. Intercropping involves the simultaneous cultivation of multiple crops (e.g., herbaceous plants, shrubs, or other plants) on the same land, leveraging the growth characteristics and functional diversity of plants to enhance resource utilization efficiency, benefiting all crops involved. Through extensive practice, various tea plantation ecological models based on composite planting have emerged in tea-producing regions, primarily encompassing tea–forest (fruit), tea–grass, and tea–forest–grass systems. Prior to the 1980 s, research on intercropping in tea plantations was limited. It was not until 2010 that foundational research in this area began to gradually increase, with initial studies focusing primarily on the impact of intercropping on tea plantation microclimates. Subsequently, intercropping models in tea plantations have developed rapidly, with associated research diversifying to address topics such as pests, soil nutrients, and microorganisms (Lei et al. 2022). Currently, the ecological functions of plant-type intercropping in tea plantations have attracted considerable attention. Compared to pure tea plantations, agroforestry ecosystems that integrate tea plants with other plant species offer distinct advantages. This model enhances biodiversity, resulting in a more complex and stable species structure, and generates mutual compensation effects, while also stabilizing the tea plantation ecosystem and increasing economic benefits (Lei et al. 2022).

*Acacia confusa* Merr., a leguminous species suitable for intercropping with tea plants, is predominantly found in Taiwan, Fujian, Guangdong, Guangxi, and Yunnan provinces of China, covering an extensive cultivation area. Compared to monoculture tea plantations, those intercropped with *A. confusa* exhibit higher biodiversity, demonstrating a significant increase in natural pest predators (e.g., spiders) and neutral insects, which substantially reduce pest populations (Li et al. 2019). The accumulation of these natural enemies and neutral insects provides effective pest control, contributing to increased tea yields. Furthermore, intercropping improves the microclimate, soil nutrient levels, and soil properties of tea plantations, thereby promoting the synthesis of quality-related components in tea leaves (Lei et al. 2022). Studies indicate that *A. confusa* intercropping enhances tea quality by increasing terpenoid contents (Li et al. 2019). While previous research has elucidated the beneficial effects of *A. confusa* intercropping on tea plantation biodiversity and tea quality, the main active components have not been thoroughly characterized in terms of their biosynthetic pathways and underlying mechanisms. Consequently, this study focuses on (*Z*)−3-hexenyl acetate, the primary volatile compound in *A. confusa*, analyzing its biosynthesis and effects on insect behavior. The findings of this study provide a scientific foundation for the effective development and implementation of plant intercropping models, with potential applications in tea plantations.

## Results

### Analysis of the primary volatile components in *A. confuse* leaves

**Fig. 1:**
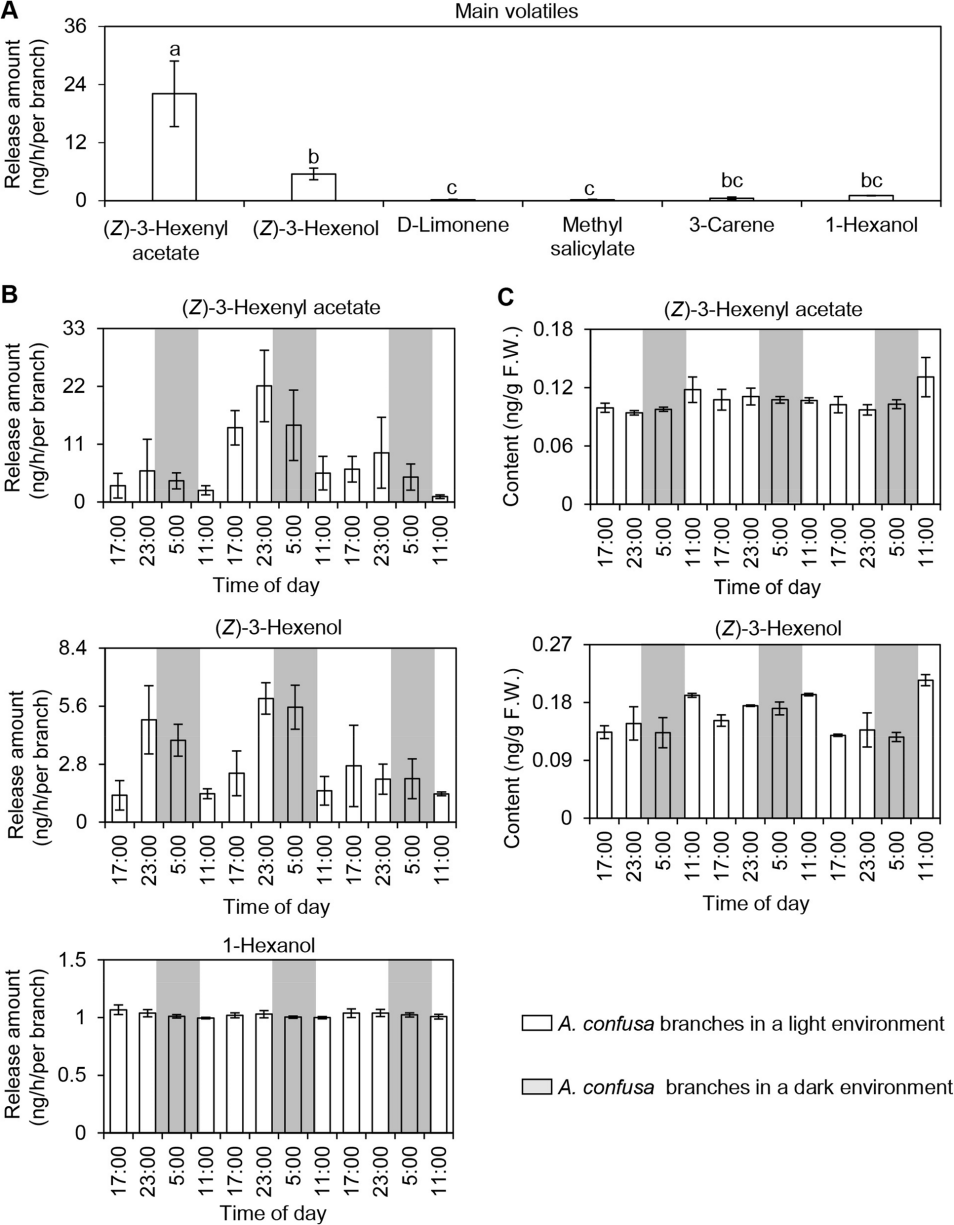
Principal volatile compounds in *Acacia confusa* Merr. and their evolution patterns over a 3-day period. **A** Principal volatile compounds of *A. confusa*. Data are presented as mean ± S.D. (*n* = 6). Significant differences between the two groups are denoted by different letters (*p* < 0.05). **B** Temporal patterns of emitted (*Z*)-3-hexenyl acetate, (*Z*)- 3-hexenol and 1-hexanol over a 3-day period. Volatiles for each replicate were collected from four branches of *A. confusa*. Data are expressed as mean ± S.D. (*n* = 6). **C** Temporal patterns of endogenous (*Z*)-3-hexenyl acetate and (*Z*)- 3-hexenol. Data are expressed as mean ± S.D. (*n* = 3). F.W., fresh weight. In B and C, white and grey backgrounds indicate day and night, respectively

In natural environments, plant volatile metabolites serve crucial ecological functions. These compounds can directly deter or attract pests and their natural predators, or act as signaling molecules that enhance the resistance of neighboring plants (Turlings et al., 2018). The primary volatile compounds responsible for the ecological effects of *A. confusa* in tea plantations remain unidentified. Consequently, analyzing the composition and emission patterns of volatiles from *A. confusa* leaves could provide valuable insights into how *A. confusa* intercropping influences insect diversity in tea plantations. The volatile components in *A. confusa* leaves primarily comprise green leaf volatiles, terpenoids, fatty acid derivatives, and alkylbenzenes/phenylpropanes compounds (Table S1). Among these volatiles, the most abundant compounds were (*Z*)−3-hexenyl acetate, (*Z*)− 3-hexenol, 3-carene, 1-hexanol, D-limonene, and methyl salicylate (Fig. 1A). Furthermore, the emission patterns of (*Z*)− 3-hexenol, 1-hexanol and (*Z*)-3-hexenyl acetate from *A. confusa* were examined using a Thermal Desorption Gas Chromatography-Mass Spectrometry (TDU-GC-MS) continuous collection and analysis system. The results indicated that (*Z*)− 3-hexenol and (*Z*)−3-hexenyl acetate exhibited a consistent emission pattern. This suggests that variations in the content of (*Z*)− 3-hexenol, the precursor of (*Z*)−3-hexenyl acetate, may influence the emission pattern of (*Z*)−3-hexenyl acetate. In contrast, the emission of 1-hexanol showed minimal variation over the same period (Fig. 1B). Additionally, analysis of endogenous volatile concentrations revealed no significant changes in the levels of (*Z*)− 3-hexenol and (*Z*)−3-hexenyl acetate in *A. confusa* (Fig. 1C), while endogenous 1-hexenol was relatively low and below the detection limit. The emission patterns of plant volatiles are known to influence the behavior of both pests and their natural enemies (Qian et al. 2024). The green leaf volatiles in *A. confusa* may be emitted rapidly after formation and function as signaling substances to participate in the defense of neighboring plants. Similarly, in maize, leaves damaged by insects rapidly emit aliphatic volatiles, including (*Z*)−3-hexenyl acetate, enhancing defenses and benefiting neighboring plants (Wang et al. 2023b).

### Analysis of primary volatile compounds in *A. confusa* for pest management in tea plantations and Y-tube olfactometer studies

**Fig. 2:**
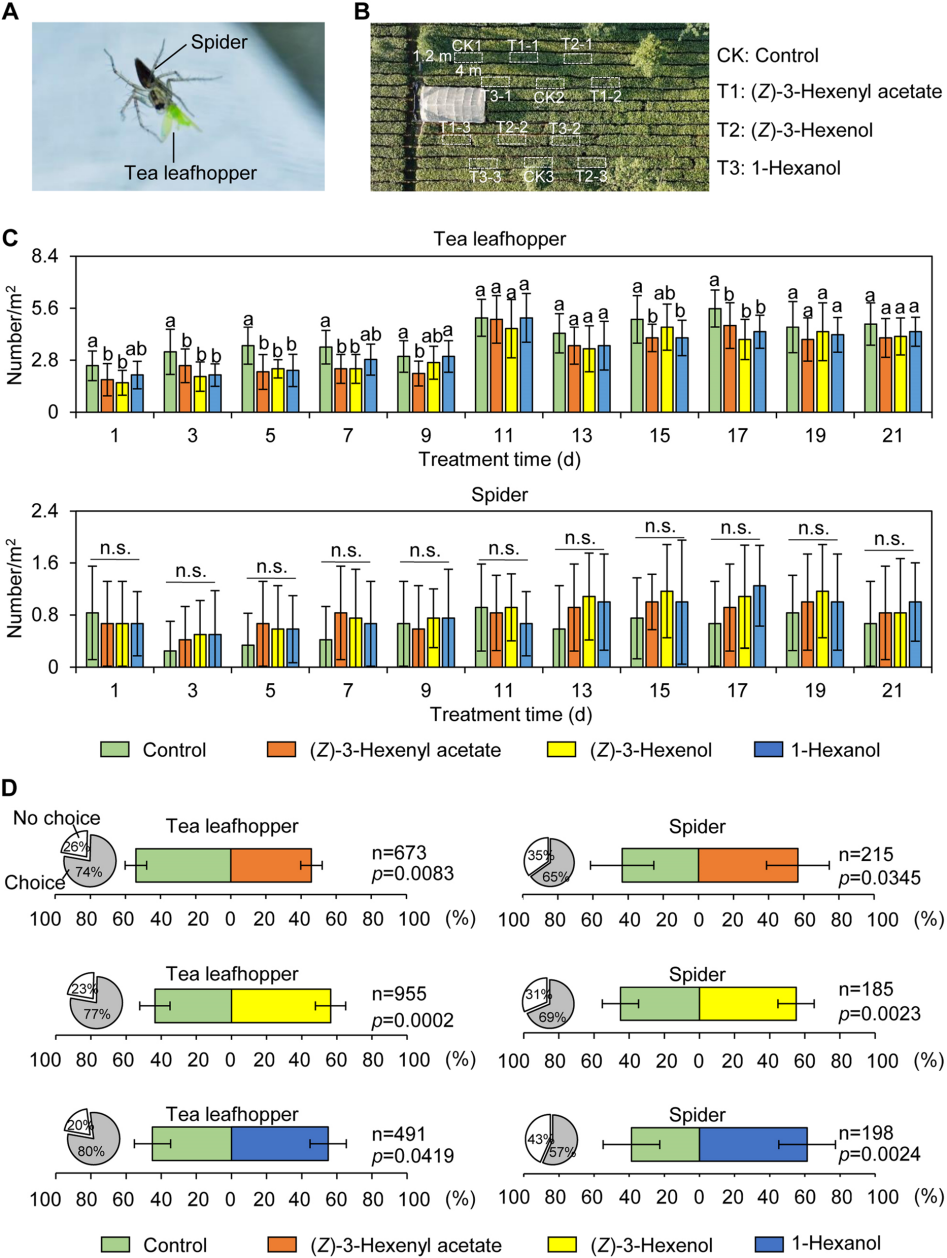
Analysis of the effects of primary volatiles on tea leafhoppers and spiders. **A** Spider predating on tea leafhopper in a tea plantation. **B** Field slow-release agents for control: (*Z*)-3-hexenyl acetate, (*Z*)- 3-hexenol, and 1-hexanol. Each group consisted of three replicates, with each replicate measuring 1.2 m × 4 m. **C** Impact of (*Z*)-3-hexenyl acetate, (*Z*)- 3-hexenol, and 1-hexanol slow-release agents on tea leafhoppers and their predatory spiders. Each treatment group comprised three replicates, with four randomly sampled quadrats (1 m^2^) surveyed per replicate. Data are presented as mean ± S.D. (*n* = 12). Significant differences between groups are indicated by different letters at the same time point (*p* < 0.05). n.s., not significant. In B and C, slow-release field experiments were conducted from 28 October to 17 November, 2024. **D** Y-tube olfactometer assay for (*Z*)-3-hexenyl acetate, (*Z*)- 3-hexenol, and 1-hexanol. Leafhopper groups: each treatment comprised a minimum of 10 replicates with at least 50 leafhoppers per replicate. Spider groups: each treatment included no fewer than 12 replicates containing a minimum of 10 spiders per replicate. Data are expressed as mean ± S.D. (*n* ≥ 185). Significant differences between groups at *p* < 0.05

Plant volatiles, as crucial defense-related compounds, may regulate pest populations in tea plantations intercropped with *A. confusa*. However, the key compounds produced by *A. confusa* that influence insect communities remain unidentified. Furthermore, while (*Z*)− 3-hexenol, 1-hexanol, and (*Z*)−3-hexenyl acetate have been identified as major volatile compounds in *A. confusa* leaves, their efficacy in pest management within tea plantations has not been evaluated. A previous study indicated that intercropping with *A. confusa* primarily affected common tea pests, including the tea leafhopper (*Empoasca onukii* Matsuda) and its natural enemy, spiders (Li et al. 2019). Consequently, this study focused on the effects of (*Z*)− 3-hexenol, 1-hexanol, and (*Z*)−3-hexenyl acetate on tea leafhoppers and spiders in tea plantation (Fig. 2A-B). The temperature and humidity during the experiment are illustrated in Fig. S1. The experimental region is characterized by a subtropical monsoon climate with an average annual temperature of 20.7 °C, average annual rainfall of 1883.9 mm, and average relative humidity of 79%. The tea leafhopper is one of the most widespread, harmful, and challenging pests to control in tea plantations (Zhang et al. 2019). Spiders, particularly Araneidae, Salticidae, Lycosa, and Oxyopes (*Xyopes sertatus* L. Koch), are the primary natural enemies of the tea leafhopper. Therefore, the impact of (*Z*)− 3-hexenol, 1-hexanol, and (*Z*)−3-hexenyl acetate, the main volatiles of *A. confusa*, on tea leafhoppers and spiders was investigated (Fig. 2A-B). Field application of slow-release (*Z*)− 3-hexenol, 1-hexanol, and (*Z*)−3-hexenyl acetate significantly reduced the number of tea leafhoppers and slightly increased the spider population (Fig. 2C). To further validate these effects, a Y-tube olfactometer experiment was conducted (Wu et al. 2023). The results revealed that (*Z*)−3-hexenyl acetate repelled tea leafhoppers, while (*Z*)− 3-hexenol and 1-hexanol attracted them. Additionally, all these volatiles were found to be attractive to tea plantation spiders (Fig. 2D). The results for (*Z*)−3-hexenyl acetate in this experiment were consistent with those observed in the June 2024 experiment (Fig. S2). Moreover, the use of (*Z*)−3-hexenyl acetate slow-release agents increased tea yield, with a significant increase in the weight of 100 tea buds (Fig. S2). In conclusion, these findings suggest that green leaf volatiles, particularly (*Z*)−3-hexenyl acetate, may be crucial active components in pest control when intercropping *A. confusa* in tea plantations.

### Analysis of major volatiles of *A. confusa* for signal transduction control of tea plant pest

**Fig. 3:**
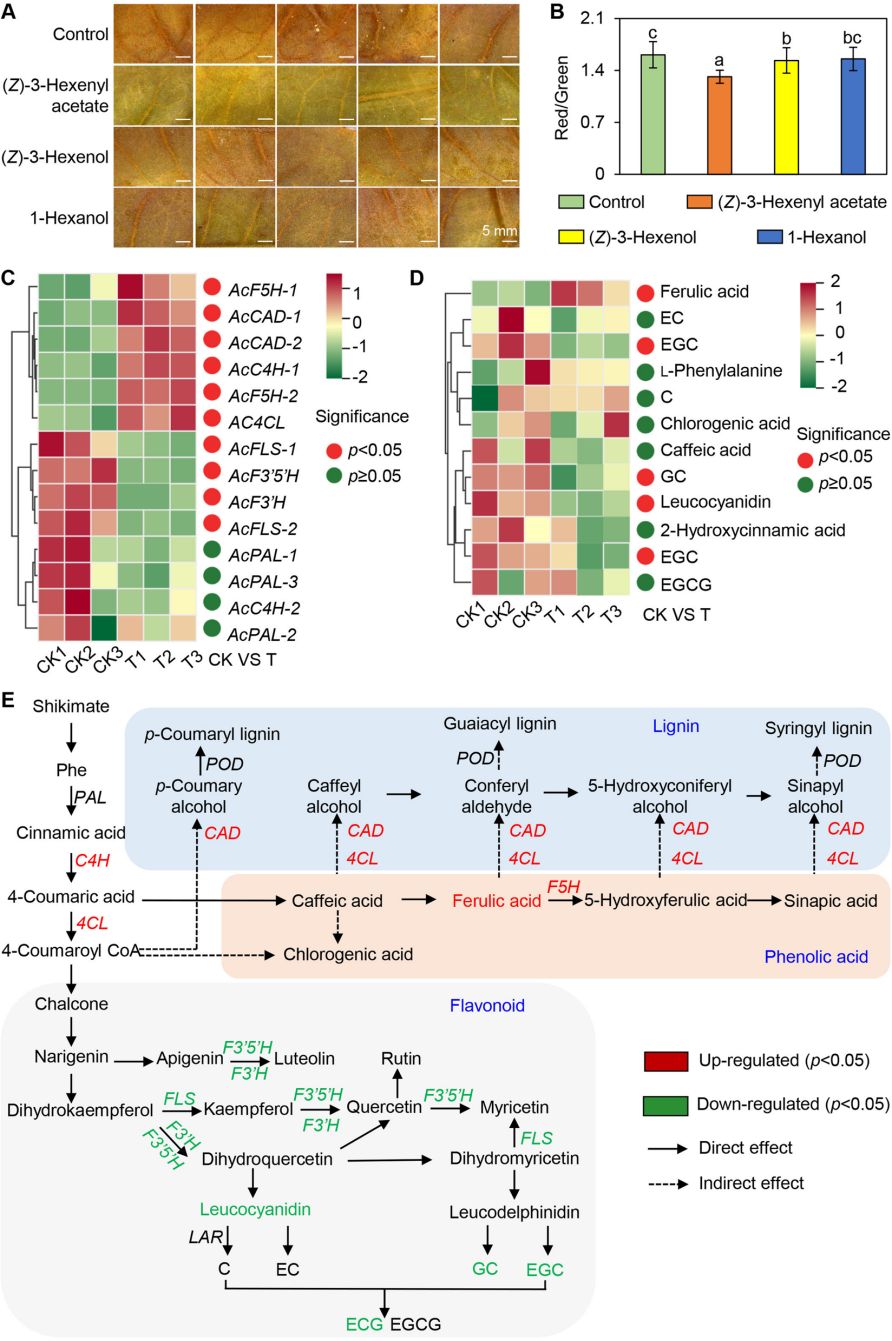
Changes in defense metabolites in tea leaves under (*Z*)−3-hexenyl acetate treatment. **A**-**B** Evaluating the degree of tea leafhopper infestation in tea leaves under volatile fumigation treatments. Original tea leaf images following leafhopper infestation are presented in Fig. S4. **A** Micrographs; **B** Quantitative analysis of foliar damage severity. Data are expressed as mean ± S.D. (*n* = 20). Each tea leaf was divided into 20 portions, and the RGB value of each portion was extracted and averaged. **C** Transcriptomic analysis of tea leaves fumigated with (*Z*)-3-hexenyl acetate. **D** Metabolomic analysis of tea leaves fumigated with (*Z*)-3-hexenyl acetate. **E** Comprehensive diagram of the metabolome and transcriptome. In C and E, significant differences between control and experimental treatment groups are indicated (*p* < 0.05). PAL, phenylalanine ammonia-lyase; C4H, phenylalanine ammonialyase; CAD: cinnamyl-alcohol dehydrogenase; POD, peroxidase; 4 CL: 4-coumarate-CoA ligase; F5H: ferulic acid- 5-hydroxylase; F3′5'H: flavonoid 3′ 5'-hydroxylase; F3'H: flavonoid 3'-hydroxylase; FLS: flavanol synthase enzyme. LAR: leucoanthocyanidin reductase.

To further assess the defensive properties of these three volatile compounds on tea leaves, a fumigation treatment was conducted. Leaf damage was evaluated by extracting the Red, Green, and Blue (RGB) values from leaves following leafhopper infestation, with the red-to-green ratio serving as an indicator of foliar injury severity (Fig. S3). The results demonstrated that (*Z*)− 3-hexenol and (*Z*)−3-hexenyl acetate significantly reduced damage caused by tea leafhoppers, with (*Z*)−3-hexenyl acetate showing particularly notable effects (Fig. 3A-B and S4). Plant volatiles can induce the production of defensive metabolites through signal transduction, thereby enhancing the defenses of both the emitting plant and neighboring plants (Jian et al. 2021). To determine whether (*Z*)−3-hexenyl acetate affects defensive metabolites in tea plants through signal transduction and to elucidate the mechanism mediating its effects on insect behavior, we fumigated tea plant branches with (*Z*)−3-hexenyl acetate, followed by transcriptome and metabolome analyses. Principal component analysis (PCA) revealed significant separation between control and treatment groups based on key features. At the transcriptome level, differentially expressed genes were predominantly associated with metabolic pathways, including starch, sucrose and glutathione metabolism, and phenylpropanoid and flavonoid biosynthesis. Additionally, genes involved in phytohormone signaling transduction and the mitogen-activated protein kinase (MAPK) signaling pathway exhibited significant changes (Fig. S5). At the metabolic level, differentially abundant metabolites were primarily related to glycerophospholipid, cysteine, methionine and phenylalanine metabolism, and pantothenate, CoA, flavone aglycones and flavonol glycoside biosynthesis (Fig. S6).

Phenolic acids, lignin, and flavonoids in plants play a crucial role in protecting plants from insect herbivory (War et al. 2012; Kumar et al. 2020). The synergistic action of these compounds may enhance plant resistance to pest attacks and mitigate damage. The results demonstrated that (*Z*)−3-hexenyl acetate significantly influenced flavonoid and phenolic acid metabolic pathways in tea leaves (Fig. 3). Further analyses revealed that (*Z*)−3-hexenyl acetate elevated the levels of defensive compounds, particularly ferulic acid (Fig. 3D). However, the (*Z*)−3-hexenyl acetate treatment significantly reduced flavonoid levels, possibly due to the diversion of common upstream substrates towards phenolic acid and lignin production rather than flavonoid synthesis (i.e., metabolic flux) (Fig. 3E). At the gene transcription level, flavonoid synthesis genes such as flavonoid 3′ 5'-hydroxylase (F3′5'H), flavonoid 3'-hydroxylase (F3'H), and flavanol synthase enzyme (FLS) were significantly downregulated. Additionally, (*Z*)−3-hexenyl acetate significantly activated secondary metabolism pathways by upregulating the expression of genes encoding enzymes involved in lignin and phenolic acid synthesis, including phenylalanine ammonia-lyase (*AcC4H*), 4-coumarate-CoA ligase (*Ac4 CL)*, ferulic acid- 5-hydroxylase (*AcF5H*) and cinnamyl-alcohol dehydrogenase (*AcCAD*), ultimately enhancing the defensive capabilities of the tea plant (Fig. 3E). These findings suggest that (*Z*)−3-hexenyl acetate may directly control pests while also functioning as a signaling molecule that promotes the accumulation of defensive metabolites, thereby stabilizing tea plant productivity.

### Analysis of the (*Z*)−3-hexenyl acetate biosynthetic pathway and identification of key candidate genes

**Fig. 4:**
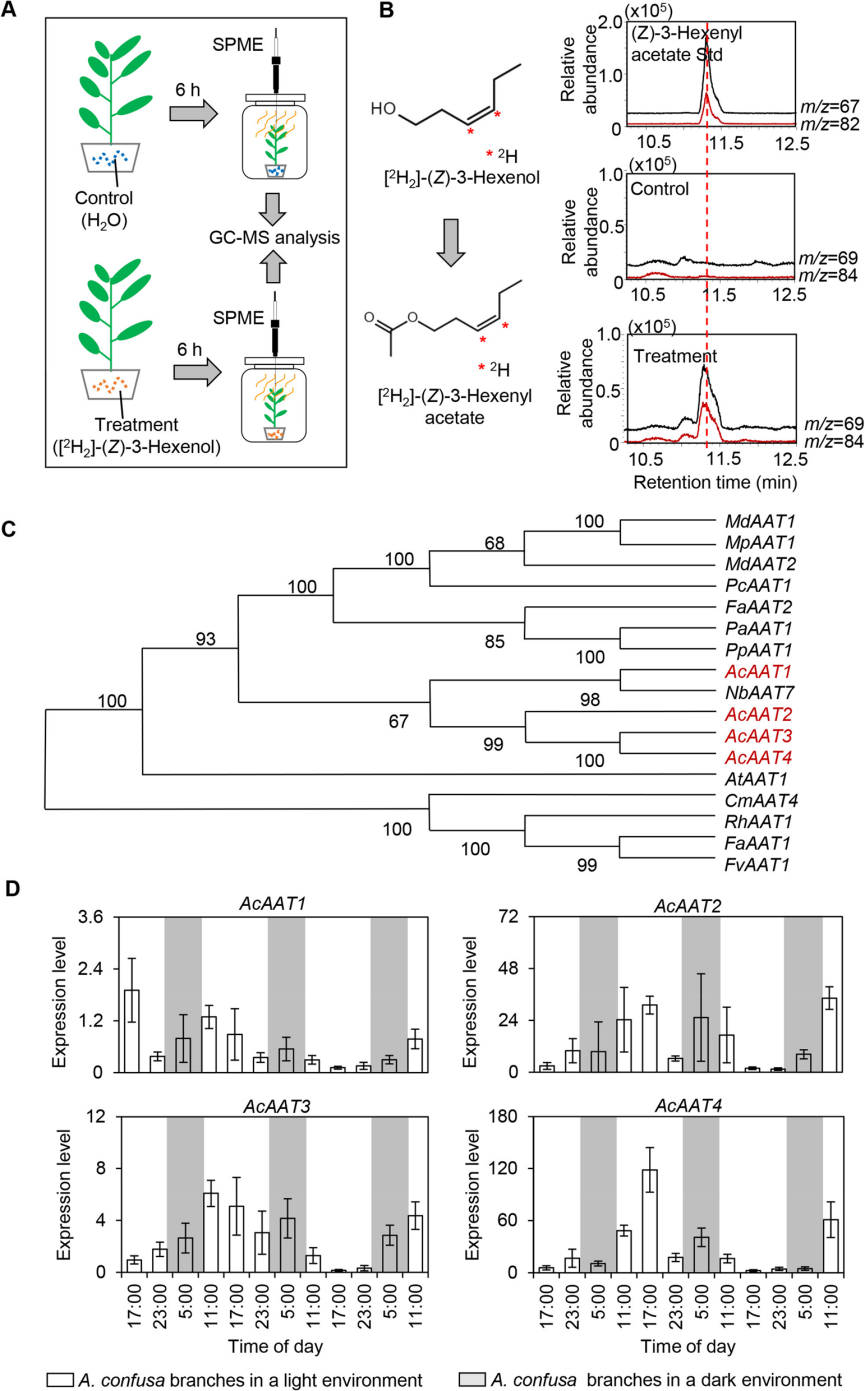
Analysis of the (*Z*)−3-hexenyl acetate biosynthetic pathway in *Acacia confusa* Merr. **A** Schematic representation of the isotope tracing experiment involving labeled (*Z*)- 3-hexenol. **B** Metabolic pathway analysis of (*Z*)- 3-hexenyl acetate. Data are presented as mean ± S.D. (*n* = 5). The comparative data are based on the relative contents of both the precursor (*Z*)- 3-hexenol and the isotope-labeled product (*Z*)- 3-hexenyl acetate in the treated and control leaves. **C** Phylogenetic analysis of (*Z*)- 3-hexenyl acetate synthesis genes. *Ac*, *A*. *confusa*; *Md*, *Malus domestica*, *MdAAT1* (Yauk et al. 2017), MdAAT2 (Li et al., 2006); *Fa*, *Fragaria ananassa*, *FaAAT1* (Aharoni et al. 2000), *FaAAT2* (Guadalupe et al. 2012); *At*, *Arabidopsis thaliana*, *AtAAT1* (D'Auria et al. 2007); *Cm*, *Cucumis melo*, *CmAAT4* (El-Sharkawy et al., 2005); *Fv, Fragaria vesca*, *FvAAT1* (Aharoni et al. 1999); *Pa*, *Prunus armeniaca*, *PaAAT1* (Mauricio et al., 2009); *Pc*, *Pyrus communis*, *PcAAT1* (Beekwilder et al., 2004); *Pp*, *Prunus persica*, *PpAAT1* (Accession number: DY645545); *Rh, Rosa hybrid, RhAAT1* (Shalit et al., 2003); *Nb, Nicotiana benthamiana, NdAAT7* (Accession number: LOC107776214)*. AcAAT*, *alcohol acyl transferase*. **D** Expression analysis of *AcAAT* in response to mechanical damage. Data are expressed as mean ± S.D. (*n* = 3). *AcAAT*, alcohol acyltransferase gene. White and grey backgrounds denote day and night, respectively

A previous study demonstrated that (*Z*)−3-hexenyl acetate is synthesized from (*Z*)− 3-hexenol through an esterification reaction catalyzed by alcohol acyltransferase (AAT), involving acyl-CoA and (*Z*)− 3-hexenol (Zhou et al., 2021). To further elucidate the (*Z*)−3-hexenyl acetate biosynthetic pathway in *A. confusa*, branches were treated with isotope-labeled (*Z*)− 3-hexenol. Analysis of treated branches revealed a significant increase in isotope-labeled (*Z*)−3-hexenyl acetate in leaves (Fig. 4A-B), suggesting that (*Z*)−3-hexenyl acetate is also synthesized from (*Z*)− 3-hexenol in *A. confusa*. The final step of the volatile ester biosynthetic pathway is catalyzed by AAT, a crucial enzyme for aroma compound formation. Through literature analysis regarding (*Z*)−3-hexenyl acetate, homology alignments, and phylogenetic analyses using *A. confusa* transcriptome data, four *AAT* genes were identified: *AcAAT1*, *AcAAT2*, *AcAAT3*, and *AcAAT4* (Fig. 4C). Quantitative PCR results indicated that mechanical damage significantly upregulated the expression of *AcAAT2*, *AcAAT3*, and *AcAAT4*. Notably, *AcAAT4* expression was significantly upregulated in response to mechanical damage and correlated with the changing trend of (*Z*)−3-hexenyl acetate content (Fig. 4D), suggesting its potential role as an important candidate gene in (*Z*)−3-hexenyl acetate synthesis.

### Functional characterization of key genes involved in (*Z*)−3-hexenyl acetate synthesis

**Fig. 5:**
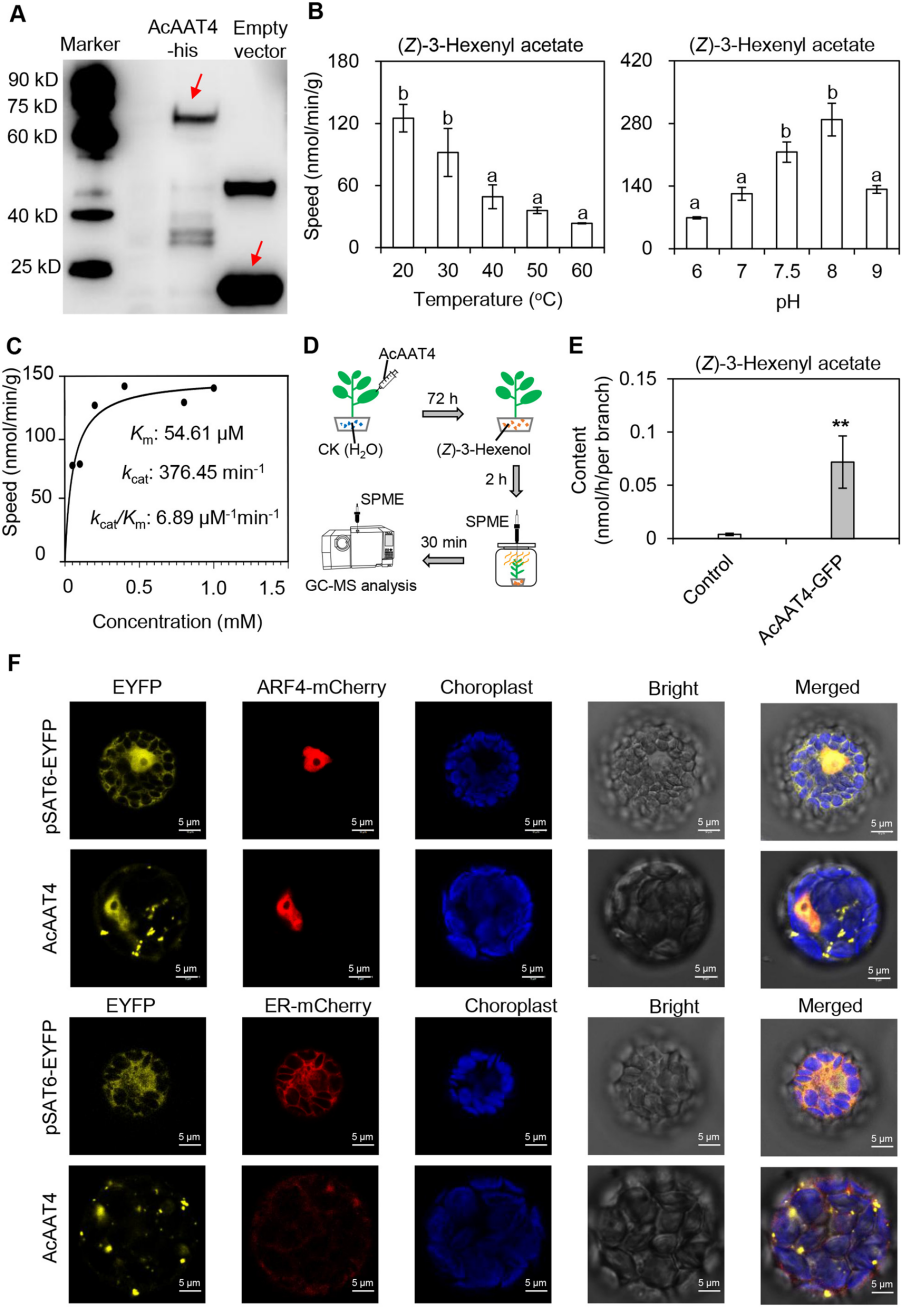
Analysis of AcAAT4 characteristics and transient expression in tobacco. **A** Western blot analysis of AcAAT4-his. **B** Impact of varying pH and temperatures on AcAAT4 activity. Data are presented as mean ± S.D. (*n* = 3). Significant differences between groups are denoted by different letters (*p* < 0.05). **C** Analysis of AcAAT4 kinetic parameters. Data are expressed as mean ± S.D. (*n *= 4). **D** Schematic of tobacco transient expression experiment. **E** GC-MS results for AcAAT4 transiently expressed in tobacco. Data are presented as mean ± standard deviation (*n *= 3). Significant differences between control and experimental groups are indicated (*, *p* < 0.05 and **, *p* < 0.01). **F** Subcellular localization of AcAAT4. Yellow fluorescence represents the AcAAT4-EYFP fusion protein, red fluorescence indicates the marker signal, and blue fluorescence shows chlorophyll autofluorescence. Data are expressed as mean ± S.D. (*n* = 3). AcAAT, alcohol acyltransferase

To identify the key genes contributing to (*Z*)−3-hexenyl acetate synthesis, recombinant AcAAT proteins were analyzed using (*Z*)− 3-hexenol and acetyl-CoA. Both AcAAT1 and AcAAT4 demonstrated the ability to produce (*Z*)−3-hexenyl acetate in vitro (Fig. S7). Notably, *AcAAT4* exhibited significantly higher expression levels than *AcAAT1* (Fig. 4D), suggesting *AcAAT4* as the more prominent candidate gene. SDS-PAGE analysis revealed the molecular mass of AcAAT4-His to be approximately 72.66 kDa (Fig. 5A). The enzymatic activity of AcAAT4 decreased with increasing temperature, resulting in a significant reduction in product formation. Additionally, AcAAT4 activity increased as pH rose from 6 to 8, but decreased markedly at pH 9. These findings indicate that AcAAT4 activity is optimal at pH 7.5–8 and 30 °C (Fig. 5B). Utilizing these optimal conditions, enzyme activity was analyzed using varying concentrations of (*Z*)− 3-hexenol substrate. The resulting kinetic parameters for AcAAT4 were: Michaelis constant (*K*m) value of 54.61 μM and *K*cat/*K*m value of 6.89 μM^−1^ min^−1^ (Fig. 5C). Due to the absence of a mature genetic transformation system for *A. confusa*, the model plant *Nicotiana benthamiana* (*N. benthamiana*) was selected for transient expression analysis to validate the in vivo function of AcAAT4. Results demonstrated that AcAAT4 significantly elevated (*Z*)−3-hexenyl acetate levels in *N. benthamiana* (Fig. 5D-E and S8), confirming the importance of *AcAAT4* in (*Z*)−3-hexenyl acetate synthesis. Subsequently, the subcellular localization of AcAAT4 was examined in *Arabidopsis*. Using nucleus-localized ARF4-mCherry and cytoplasm-localized ARF4-mCherry as controls, AcAAT4 was observed to localize in both the nucleus and cytoplasm (Fig. 5F).

### Mechanism regulating (*Z*)−3-hexenyl acetate biosynthesis

**Fig. 6:**
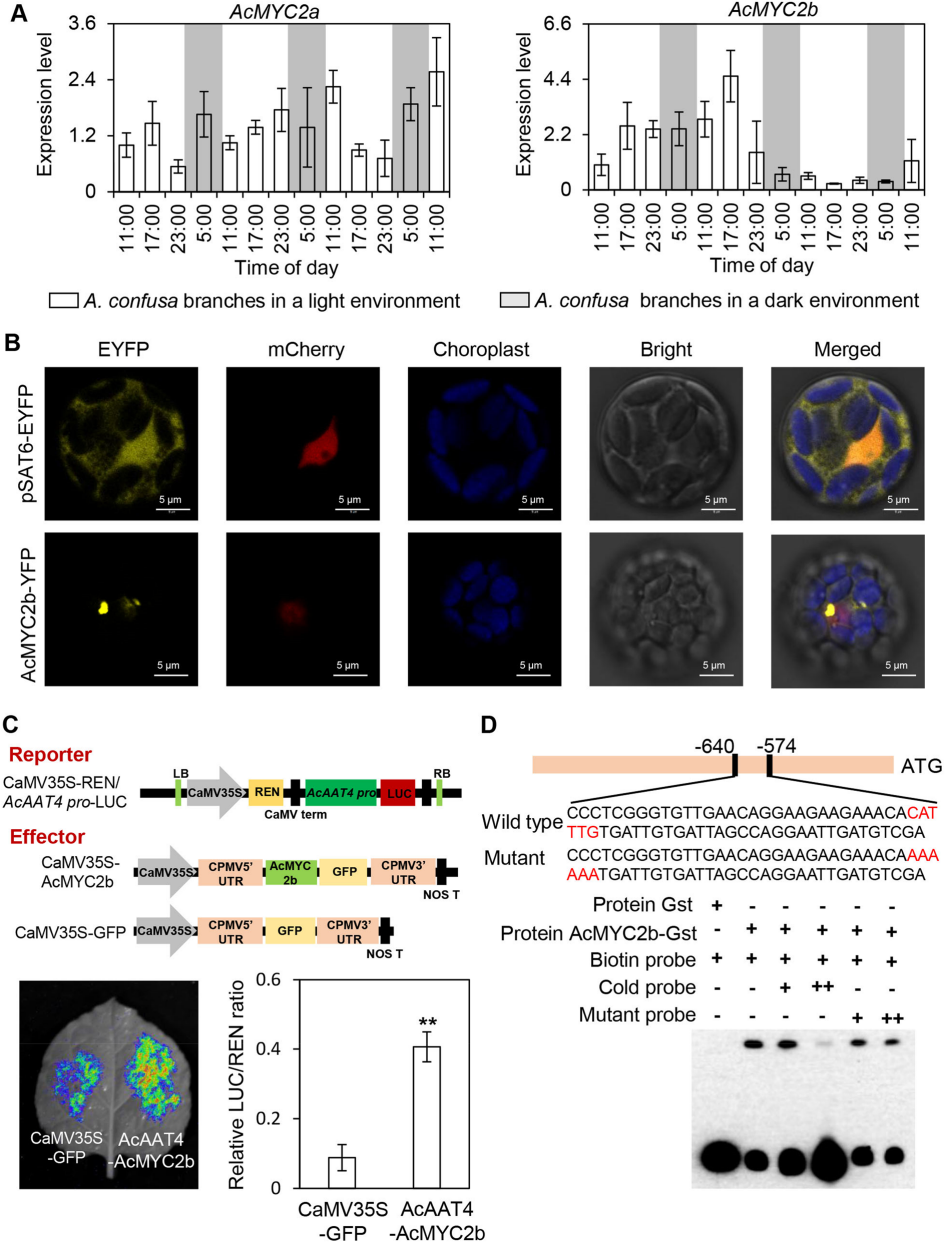
Analysis of the interaction between AcMYC2 and *AcAAT4. ***A** Analysis of *AcMYC2* expression in response to continuous mechanical damage. White and grey backgrounds indicate day and night, respectively. **B** Subcellular localization of AcMYC2. Yellow fluorescence represents the AcAAT4-EYFP fusion protein, red fluorescence indicates the marker signal, and blue fluorescence denotes chlorophyll autofluorescence. **C **Analysis of transcriptional activation in tobacco leaves. In A, B and C, data are presented as mean ± S.D. (*n* = 5). Significant differences between control and experimental treatment groups are indicated (*, *p* < 0.05 and **, *p* < 0.01). **D** Verification of AcAAT4 interactions with AcMYC2b by EMSA. AcAAT, alcohol acyltransferase; AcMYC2, myelocytomatosis protein 2; EMSA, electrophoretic mobility shift assay

Gene expression is regulated at the transcriptional level, but the transcription factors governing *AcAAT4* expression have remained unidentified. Jasmonic acid (JA), a plant hormone crucial for defense responses and development, has been implicated in the regulation of plant volatile synthesis (Wang et al. 2020; Li et al. 2024). The MYC2 transcription factor, extensively studied and functionally potent, is a key regulator of the JA signaling pathway in plants (Wang et al. 2020). Based on MYC2, we identified AcMYC2a and AcMYC2b as potential transcription factors in *A. confusa* that may regulate volatile compound synthesis under pest-related stress. Phylogenetic analysis revealed that these two MYC2 transcription factors are highly similar and closely related to MYC2 from rice, *Arabidopsis*, and walnut (Fig. S9). The expression of *AcMYC2b* increased significantly during the first 2 days of continuous mechanical damage-induced stress, aligning with *AcAAT4* expression levels (Fig. 6A). Subcellular localization analysis demonstrated AcMYC2b's presence in the cell nucleus (Fig. 6B), suggesting its role as a transcription factor. Transcriptional activation experiments revealed that AcAAT4-AcMYC2b treatment significantly enhanced the fluorescence signal compared to the control group (Fig. 6C). Protein extract analysis showed a marked increase in the LUC/REN ratio (Fig. 6C), demonstrating AcMYC2b's ability to significantly activate *AcAAT4* expression. Additionally, Electrophoretic mobility shift assay (EMSA) results confirmed the interaction between AcMYC2b and *AcAAT4* (Fig. 6D). These findings suggest AcMYC2b as a key regulator of (*Z*)−3-hexenyl acetate synthesis. Consequently, *AcAAT4* expression appears to be the primary factor mediating (*Z*)−3-hexenyl acetate release, with AcMYC2b serving as a key regulator of *AcAAT4* expression. This research provides novel insights into the regulatory mechanism controlling (*Z*)−3-hexenyl acetate synthesis and its effects on plant–insect interactions.

## Discussion

### Ecological effects of (*Z*)−3-hexenyl acetate in *A. confuse* leaves

Plants synthesize a diverse array of complex volatiles, primarily categorized as fatty acid derivatives, terpenoids, and phenylpropanoids based on their biosynthetic pathways and structural characteristics (Zeng et al. 2019). These volatiles play crucial roles in plant defense mechanisms, attracting natural predators of insect pests and participating in signal transduction pathways (Zeng et al. 2019; Jin et al., 2023). A recent investigation revealed that (*Z*)−3-hexenyl acetate, a volatile released by maize in response to herbivore attacks, functions as an attractant (Aguirre et al. 2023). This compound has been shown to attract various pests, including *Agrilus planipennis*, *Agrilus mali* Matsumura, and *Plutella xylostella* (Li et al. 2012; Zhou et al. 2020a). Furthermore, (*Z*)−3-hexenyl acetate has been demonstrated to influence the host selection and oviposition behavior of *Spodoptera frugiperda* by binding to the olfactory receptor SfruOR23 (Wang et al. 2023a). Our insect resistance field experiments indicated that (*Z*)−3-hexenyl acetate can also serve as an attractant for spiders that prey on tea leafhoppers, common tea plantation pests, thereby mitigating the damage caused by these pests (Fig. 2C and S2). Additionally, Y-tube selection experiments revealed that (*Z*)−3-hexenyl acetate exhibits a repellent effect on tea leafhoppers (Fig. 2D and S2). Given the diverse effects of volatile compounds on different insects, it is essential to comprehensively evaluate the impact of a single compound on both pests and their natural enemies in small agricultural ecosystems characterized by high species diversity and complex environments.

Plant volatiles can induce defense responses *via* signal transduction, ultimately leading to the synthesis of defense-related metabolites. In maize, (*Z*)−3-hexenyl acetate increases the abundance of defense hormones, such as abscisic acid and JA. It significantly enhances the formation of defense-related metabolites, including indole, (*3E*)−4,8-dimethyl-1,3,7-nonatriene (DMNT) and (*E,E*)−4,8,12-trimethyl-1,3,7,11-tridecatetraene (TMTT), which protect plants from herbivorous pests (Aguirre et al. 2023; Wang et al. 2023b). In wheat, (*Z*)−3-hexenyl acetate modulates plant defenses through the production of reactive oxygen species, activation of antioxidant pathways, and induction of glycosylation (Ameye et al. 2020). In tea plants, (*Z*)−3-hexenyl acetate serves multiple functions in defense against herbivores, not only directly participating in defense but also enhancing overall defense capabilities by regulating internal signaling and hormone levels, as well as promoting plant-plant communication (Gu et al 2024). Exogenous treatment with (*Z*)−3-hexenyl acetate significantly elevates the levels of JA and JA-Ile, and the expression levels of JA signaling related synthesis genes such as *CsLOX1*, *CsLOX3*, *CsPI1*, *CsADH2* and *CsHPL* increase substantially, thereby enhancing the defensive capacity of tea leaves against *Ectropis obliqua* (*E. obliqua*) infestation. By suppressing the expression of the (*Z*)−3-hexenyl acetate synthesis gene *CsCHAT1*, it was observed that the accumulation of (*Z*)−3-hexenyl acetate decreased and the resistance of tea plants to *E. obliqua* diminished. In addition, the transcription factors *CsNAC30* and *CsTCP11* positively regulate the expression of *CsCHAT1* and were involved in the defense response of tea plants to herbivore attack (Gu et al 2024). In the current study, we observed that the ferulic acid level increased in tea leaves following the fumigation with exogenous (*Z*)−3-hexenyl acetate (Fig. 3 and S4). Phenolic acids are crucial for plant resistance and defense against insect herbivory. In rice, the levels of phenolic acids, including cinnamic acid, vanillic acid, coumaric acid, and ferulic acid, increase significantly following an attack by insects, with the extent of the increase correlated with the severity of the herbivore-induced damage (Kelly et al., 2002; Usha et al., 2009). At the transcriptional level, (*Z*)−3-hexenyl acetate significantly upregulated the expression of the genes involved in the biosynthesis of the upstream metabolites 4-coumaric acid (C4H) and 4-coumaroyl-CoA (4 CL) (Fig. 3C). Additionally, we detected a significant increase in the expression of the lignin biosynthesis-associated genes *CAD* and *4 CL* (Fig. 3C-E). Upregulated expression of these genes promotes lignin deposition, thereby enhancing plant physical defenses (Yadav et al., 2023). In rice, the transcription factors MYB30, MYB55, and MYB110 induce ferulic acid accumulation by regulating genes in the cinnamic acid and lignin biosynthesis pathways (Kishi-Kaboshi et al. 2018). In addition, the transcription factor CmMYC15 enhances the resistance of chrysanthemum to aphid attack by activating lignin synthesis-related genes (An et al., 2019). In the present study, the abundance of flavonoids, especially catechins, and the expression of flavonoid biosynthesis genes decreased significantly after the (*Z*)−3-hexenyl acetate treatment. This may be attributed to metabolic flux, with precursors being directed towards the synthesis of phenolic acids and lignin, consistent with the upregulated expression of genes related to lignin and phenolic acid biosynthesis (Fig. 3E). In conclusion, due to its molecular effects, (*Z*)−3-hexenyl acetate may be valuable in the development of improved pest control strategies.

### Biosynthesis of (*Z*)−3-hexenyl acetate in *A. confuse* leaves

Plant volatile esters play critical roles in flavor formation, interplant signaling, and biotic stress responses. AATs are considered to catalyze the rate-limiting step in the synthesis of these ester volatiles (Sharma et al., 2013). These enzymes, belonging to the BAHD superfamily, transfer acyl groups from CoA donors to alcohol acceptors to form ester compounds (Souleyre et al. 2014). The substrate specificity of AATs determines the specific esters they can synthesize (Cumplido-Laso et al., 2012). Through screening and functional characterization of genes, we identified AcAAT4 as the key enzyme catalyzing the formation of (*Z*)−3-hexenyl acetate from acetyl-CoA and (*Z*)− 3-hexenol (Fig. 5A-E). AcAAT4 in *A. confusa* demonstrates a high affinity for (*Z*)− 3-hexenol, with a *Km* of 54.61 μM (Fig. 5C); its catalytic efficiency exceeds that of AATs from *Arabidopsis* (*Km* of 251 μM), strawberry (*Km* of 1.53 mM), and apricot (*Km* of 1.32 mM) (D'Auria et al. 2007; Cumplido-Laso et al., 2012; Zhou et al., 2021). Thus, AcAAT4 exhibits significant catalytic advantages over similar enzymes in other species, underscoring its biological importance in the synthesis of plant volatile esters. Due to the absence of an established genetic transformation system, this study initially employed a *N. benthamiana* transient expression assay to demonstrate that AcAAT4 significantly enhances the formation of (*Z*)−3-hexenyl acetate in vivo (Fig. 5D-E). Additionally, we attempted to verify the function of *AcAAT4* using the Antisense oligonucleotides (AsODNs) system. The results indicate that the success rate of inhibition using this system is approximately 60%. Despite continuous attempts, the success rate of inhibition has not improved. Furthermore, analysis of the metabolic content and gene expression levels of the samples after inhibition revealed that inhibition of the *AcAAT4* gene significantly reduced the content of the product (*Z*)−3-hexenyl acetate in *A. confuse* (Fig. S10). This result partially confirms the function of *AcAAT4 *in vivo. However, the in vivo function of this gene requires further verification using a more stable system in future studies.

The biosynthesis of (*Z*)−3-hexenyl acetate is induced by external stresses, and the generated compound is rapidly released after formation. However, in this study, the pattern of (*Z*)−3-hexenyl acetate release differed from the endogenous (*Z*)−3-hexenyl acetate levels (Fig. 1). This discrepancy may be attributed to the properties of volatiles and transport proteins. The physicochemical properties of various volatiles differ. For instance, non-oxidized monoterpenes, such as linalool, can be stably stored in leaves due to their high octanol/water partition coefficients (Loreto et al., 1998). Conversely, oxidized volatiles, including (*Z*)−3-hexenyl acetate, are likely to be released rapidly as they cannot be stored in cells for extended periods. Additionally, there is a delay between the synthesis and release of volatiles (Niinemets et al. 2014). Furthermore, various transporters, such as lipid transfer proteins and members of the ABC transporter family, regulate the release of volatiles (Liao et al. 2023; Adebesin et al. 2017).

Plant volatiles exhibit specific release patterns influenced by various factors, including environmental conditions (e.g., light and temperature), circadian rhythms, and plant and pest species (Qian et al. 2024). These patterns often display distinct diurnal rhythms. Several volatiles, such as DMNT, α-farnesene, benzyl nitrile, benzyl alcohol, indole, and TMTT, are emitted more abundantly during daytime than at night (Zhang et al. 2010; Clavijo McCormick et al., 2014; Cai et al. 2014; Qian et al. 2023; Li et al. 2024). Conversely, other volatiles, like methyl benzoate, are released at higher levels nocturnally (Kolosova et al. 2001). Plant volatiles significantly impact plant–insect interactions by attracting natural enemies of pests and influencing pest behaviors to reduce pest-induced damage (Turlings et al., 2018). Some plants release specific volatiles diurnally or during peak insect feeding activity to attract natural enemies. Volatile emissions can modulate plant defense responses to insects, directly disrupting larval feeding and influencing adult oviposition, while also providing cues for parasitoids and predators to locate prey (Qian et al. 2024). Furthermore, the dynamic rhythms of plant volatile emission, which coincide with pollinator or foraging insect activities, ensure effective transmission of pollination signals (Qian et al. 2024; Turlings et al., 2018). The release of volatiles is regulated by both intrinsic factors (hormones and circadian rhythms) and environmental factors (Qian et al. 2024).

### Mechanisms regulating the biosynthesis of (*Z*)−3-hexenyl acetate in *A. confuse* leaves

The synthesis and emission of plant volatiles are influenced by various environmental factors, including diurnal rhythms and mechanical damage (Jian et al. 2021). In these processes, precursors, genes encoding biosynthesis-related enzymes, and transcription factors play crucial roles in the metabolic pathway (Qian et al. 2024). The present study demonstrates coordinated fluctuations in precursor (*Z*)− 3-hexenol content and *AcAAT4* expression levels with (*Z*)−3-hexenyl acetate accumulation, suggesting that the biosynthesis of (*Z*)− 3-hexenol and the transcriptional regulation of *AcAAT4* may directly modulate the formation and dynamic variation of (*Z*)−3-hexenyl acetate (Fig. 1B and Fig. 4D). While *AcAAT4* has been confirmed as the gene responsible for (*Z*)−3-hexenyl acetate synthesis, other regulatory genes may potentially influence its biosynthesis. For instance, alterations in the expression levels of precursor synthesis genes, such as those regulating (*Z*)− 3-hexenol production, could affect the ultimate accumulation of this ester compound.

Upon experiencing mechanical damage, plants produce JA, which activates downstream response pathways and induces the synthesis and release of defensive volatiles (Miller et al., 2005). Previous research has identified MYC2 as a key transcription factor in the JA pathway (Dombrecht et al. 2007). Furthermore, MYC2 extensively regulates aroma-related metabolism by influencing the formation of terpenoids, phenylpropanoids, and fatty acid derivatives. MYC2 modulates the expression of terpene synthesis genes, enhancing sesquiterpene formation (Hong et al. 2012). Additionally, MYC2 has been shown to regulate indole production in tea plants by controlling the expression of the indole biosynthesis-related genes (Zhou et al. 2020b). In apple, MdMYC2 directly binds to the *MdAAT1* promoter, increasing transcription and promoting the synthesis of aroma-associated esters (Li et al. 2023). Similarly, we observed changes in the amount of (*Z*)−3-hexenyl acetate released, the expression of *AcAAT4*, and variations in *AcMYC2b* expression (Fig. 6A), suggesting that *AcAAT4* expression may be regulated by AcMYC2. Transcriptional activation assays demonstrated that AcMYC2b significantly activated the expression of *AcAAT4* (Fig. 6C). These findings suggest that AcMYC2b controls the synthesis of (*Z*)−3-hexenyl acetate by regulating *AcAAT4* expression. Moreover, while MYC2 regulates the downstream (*Z*)−3-hexenyl acetate biosynthesis genes, *MYC2* expression is itself regulated by upstream transcription factors. MYC2, along with JASMONATE-ZIM DOMAIN proteins, phytochrome-interacting factors, the circadian clock-related transcription factor TIME FOR COFFEE, and the circadian clock protein LATE ELONGATED HYPOCOTYL, collectively regulate the synthesis and emission of volatiles (Fern´andez-Calvo et al., 2011; Shin et al. 2012; Parveen et al. 2019; Zhao et al. 2021). Consequently, further research is necessary to identify and characterize the upstream regulators of AcMYC2b.

## Conclusion

**Fig. 7:**
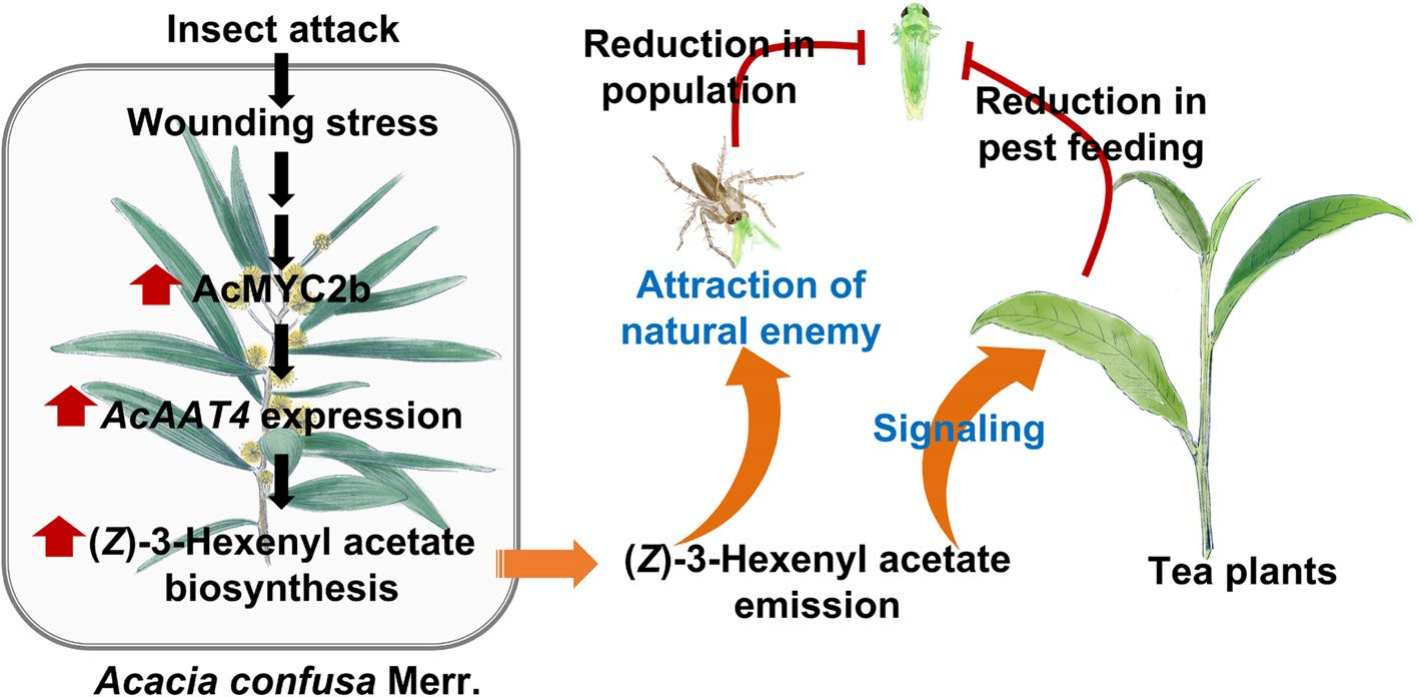
Biosynthesis of the anti-insect volatile (*Z*)−3-hexenyl acetate in *Acacia confusa* Merr. Insect herbivory induces the upregulation of the jasmonate-responsive transcription factor AcMYC2b, which activates the transcription of *AcAAT4*, a gene encoding a crucial alcohol acyltransferase. This enzymatic pathway facilitates the synthesis of (*Z*)- 3-hexenyl acetate, a volatile organic compound (VOC) that serves dual ecological purposes: (1) acting as an indirect defense signal to attract predatory spiders, and (2) functioning as a signaling molecule to enhance systemic defense responses in adjacent tea plants. This VOC-mediated tri-trophic interaction establishes an effective defense network against *Empoasca onukii* Matsuda, a predominant tea pest. AcAAT, alcohol acyltransferase; AcMYC2, myelocytomatosis protein 2

This study qualitatively identified the volatile compounds of *A. confusa* and quantitatively analyzed the major volatile compounds. The release patterns of the three primary compounds—(*Z*)− 3-hexenol, 1-hexanol, and (*Z*)−3-hexenyl acetate—were investigated. The study evaluated the biological functions of these volatiles and found significant effects on insect behavior. Among the three main volatiles in *A. confusa* leaves, (*Z*)−3-hexenyl acetate demonstrated the strongest signaling defense against tea leafhopper. Consequently, it is hypothesized that (*Z*)−3-hexenyl acetate may be a crucial active component in pest control when intercropping *A. confusa* in tea gardens (Fig. 7). The mechanism involves insect attacks activating *AcMYC2b* expression, with the encoded transcription factor subsequently enhancing *AcAAT4* expression, leading to increased (*Z*)−3-hexenyl acetate biosynthesis. Transcriptional activation and EMSA experiments confirmed the interaction between AcMYC2b and *AcAAT4*. Moreover, the expression of *AcMYC2b* and *AcAAT4* may be integral to the release of (*Z*)−3-hexenyl acetate. Previous studies have reported that intercropping with *A. confusa* can enhance biodiversity in tea plantations, notably increasing arthropod populations, including spiders. The current study revealed that plant-released (*Z*)−3-hexenyl acetate attracts spiders and other natural enemies of insect pests, effectively controlling pests while also acting as a signaling molecule that enhances neighboring tea plants' defenses against herbivorous pests (Fig. 7). This research elucidates the complex interactions between *A. confusa* and its environment, highlighting its potential for improving agricultural production.

## Materials and methods

### Experimental reagents

(*Z*)−3-hexenyl acetate was obtained from Sigma-Aldrich Company Ltd. (USA). Cellulase ‘ONOZUKA’ R10 and Macerozyme R10 were acquired from Yakult Pharmaceutical Ind. Co. (Japan). The plant RNA Rapid Extraction Kit was sourced from Huayueyang Biotechnology Co. Ltd. China. Fluorescent dye was procured from Bio-Rad Laboratories (CA, USA). Primer Script RT with gDNA Eraser, in-fusion HD cloning kit, and PrimerSTAR Max DNA polymerase were all obtained from Takara Bio Inc. (Japan). Solid phase microextraction (SPME) fibers were acquired from Supelco (DVB/Carboxen/PDMS Stable Flex, USA).

### Experimental materials

Tea plants (*C. sinensis* Yinghong No. 9), *A. confusa* and insect specimens utilized in the experiments were sourced from the Tea Research Institute, Guangdong Academy of Agricultural Sciences. *A. confusa*, a member of the Leguminosae family and Acacia genus, is a prevalent plant in South China and is frequently intercropped in tea plantations. The common pest tea leafhopper and its predatory spiders were collected from the Yingde tea plantation. *N. benthamiana* and *Arabidopsis thaliana* were cultivated in the laboratory plant room under the following conditions: 25 °C, 50–60% humidity, a photoperiod of 16 h light/8 h dark, with a light intensity of 8500 Lx.

### Analysis of the effects of main volatiles on the behavior of insects

#### Field experiment with slow-release agents

This experiment was conducted in Yingde City, Guangdong Province, China, at an altitude of 38–40 m. The tea plant variety used was *C. sinensis* Yinghong No. 9. Slow-release agents were prepared following the method described by Qian et al. (2023). A 1.5% sodium alginate solution was used to prepare (*Z*)−3-hexenyl acetate, (*Z*)− 3-hexenol and 1-hexanol solution (50 μg/g, dissolved in 1% ethanol), which was then homogenized and dropped into a 5% w/v CaCl_2_ solution to form white agents. These agents were placed in non-woven filter bags, each containing 12 g of agents. The control group contained ddH_2_O (with 1% ethanol added). Each treatment was replicated three times, with each replication covering an area of 1.2 m × 4 m, and four bags of slow-release agents were suspended per replication. Insect numbers were recorded every two days.

#### Effects of exogenous volatiles on the behavior of insects

The common tea leafhopper pest and its predator spiders were selected for this analysis using a Y-tube olfactometer. The experimental groups were subjected to 50 μL of various solutions, including 10 mM (*Z*)−3-hexenyl acetate, 10 mM (*Z*)− 3-hexenol, and 10 mM 1-hexanol, with 1% (v/v) ethanol added to enhance solubility. The control group received water containing 1% (v/v) ethanol. For the leafhopper groups, each treatment consisted of a minimum of 10 replicates, with at least 50 leafhoppers per replicate. In the spider groups, each treatment comprised no fewer than 12 replicates, containing a minimum of 10 spiders per replicate. Each individual insect was considered as one repetition.

#### Assessment of tea leaf infestation level following volatile fumigation against tea leafhoppers

The branches were pruned, retaining the second leaf, which was placed in a 5 L glass container for fumigation treatment with volatile compounds for 24 h. Treatment groups included 2 μM (*Z*)−3-hexenyl acetate, 2 μM (*Z*)− 3-hexenol, and 2 μM 1-hexanol, respectively. An equivalent volume of dichloromethane served as a control. The fumigated tea branches were subsequently placed in floral foam within wide-mouth plastic cups, with two branches per cup. Twenty tea leafhoppers were introduced to each plastic cup and incubated for one day. Each group consisted of 20 replicates. Post-infestation, the second leaf was fixed with FAA fixative solution, and the underside of the infested leaf was photographed using a stereomicroscope (Leica M205 FA, Wetzlar, Germany). The R, G, and B values of the damaged leaf color were statistically extracted using a program titled "Method for Quantifying Leaf Damage in Tea Leaves." The specific code for this method is provided in the supplementary material. The extent of leaf damage was quantified as the ratio of red value to green value (Clement et al. 2015). A ratio exceeding 1 indicated a redder coloration, signifying a higher degree of damage to the tea leaves.

### Tea plants fumigated with (*Z*)−3-hexenyl acetate

Tea branches (one bud and two leaves) were sealed in a glass jar (5 L) and exposed to 2 μM (*Z*)−3-hexenyl acetate solutions (prepared in dichloromethane). The control group received dichloromethane treatment only. The solution was applied to cotton balls (500 μL/12 h) for a total of 48 h. Each treatment was repeated 3 times, with each repetition comprising 5 branches. Metware Biotechnology Co., Ltd. (Wuhan, China) conducted the transcriptome sequencing and metabolome analysis.

### Transcriptomic analysis of tea plants fumigated with (*Z*)−3-hexenyl acetate

RNA was extracted from the samples, followed by the construction of RNA libraries and sequenced using the Illumina platform. The resulting Clean Data were aligned to the designated reference genome to obtain mapped data (Zhang et al. 2021). To quantify the expression levels of transcripts or genes, FPKM (Fragments Per Kilobase of transcript per Million fragments mapped) was utilized as the metric. The values presented in heatmaps underwent normalization using the Z-score formula. The clean read data have been deposited in the National Genomics Data Center under the accession number CRA022899.

### Metabolomic analysis of tea plants exposed to (*Z*)−3-hexenyl acetate

Samples underwent freeze-drying with liquid nitrogen, and 50 mg of fresh sample was precisely weighed. The samples were then subjected to vortex extraction following the addition of 1.2 mL of pre-cooled 70% methanol. The extracts were centrifuged at 12,000 rpm for 3 min. The resultant supernatant was filtered through a 0.22 μm membrane and subsequently analyzed using ultra-performance liquid chromatography (UPLC) (ExionLC™ AD, https://sciex.com.cn/) and tandem mass spectrometry (MS/MS). Chromatographic separation was executed on an Agilent SB-C18 column (2.1 mm × 100 mm) utilizing a mobile phase of ultrapure water containing 0.1% formic acid (A) and acetonitrile with 0.1% formic acid (B). The gradient elution commenced at 5% B, increased linearly to 95% over 9 min, maintained for 1 min, then decreased to 5% from 10.00 to 11.10 min, and equilibrated at 5% until 14 min. The flow rate was set at 0.35 mL/min, the column temperature was sustained at 40 °C, and the injection volume was 2 μL. Electrospray ionization (ESI) was performed at 550 °C with an ion spray voltage of 5500 V (positive mode) and − 4500 V (negative mode). Metabolite identification and quantification were executed based on the self-built MetWare database and the secondary mass spectrometry data of the samples.

### Analysis of the emission pattern of exogenous volatiles using TDU-GC–MS

To analyze the emission pattern of exogenous (*Z*)−3-hexenyl acetate, (*Z*)− 3-hexenol and 1-hexanol, four branches (each containing eight leaves) were placed in a sealed glass container, constituting one experimental replicate, with six replicates in total. Adsorption tubes were replaced every 6 h, and a 3 mm hole was created in each leaf using a hole puncher. Volatiles were collected using Carbon 100 columns, which were replaced with new tenax columns every 6 h, continuously collecting for 72 h with eight replicates. The collected volatiles were analyzed using TDU-GC–MS. The GC–MS model employed was an Agilent 8890/7000D Triple Quadrupole (Agilent Technologies Inc., CA, USA), equipped with a SUPELCOWAX 10 column (30 m × 0.25 mm, df 0.50 μm, Sigma-Aldrich Co. LLC, USA). The GC inlet operated in split mode (10:1) and was maintained at 200 °C for 1 min using helium carrier gas at a 1.0 mL/min flow rate. The gas chromatograph initial temperature was set at 60 °C for 3 min, then increased to 240 °C at a rate of 4 °C/min and held at 240 °C for 15 min. Mass spectrometry analysis was conducted in full scan mode over the range of *m/z* 40–200.

### Isotope tracing experiment and analysis of labeled product

The isotope tracing experiment was conducted as follows: the treatment group cultivated branches (six leaves) in a solution containing 10 μg/mL [^2^H_2_]-(*Z*)− 3-hexenol mixture, while the control group cultivated branches in pure water. All samples were incubated at 25 °C, 60% humidity, and under normal photoperiod conditions. Volatiles were collected using SPME for 1 h after 6 h post-treatment. Each group comprised three replicates. SPME samples were analyzed *via* GC–MS, with the instrument parameters consistent with those previously described.

### Phylogenetic tree analysis and transcription level analysis

Through a review of literature related to (*Z*)−3-hexenyl acetate, four *AATs* synthetic gene sequences were identified in the transcriptome data of *A. confusa* using homologous alignment. Phylogenetic analysis was conducted using MEGA software on these four candidate genes and genes reported in other plants. Transcript level analysis was performed using quantitative PCR. RNA was extracted from *A. confusa* samples utilizing an RNA extraction kit, and cDNA was synthesized using a Takara reverse transcriptase kit. Specific primers are listed in Table S2. Gene expression levels were analyzed by quantitative real-time PCR (qPCR) with SYBR green fluorescent dye. The methodology followed the protocol reported by Jian et al. (2021).

### Prokaryotic expression and in vitro enzyme activity assay

The *AcAAT4* gene was cloned into pET32a-His and transformed into *E. coli* BL21 competent cells. The relevant primers are presented in Table S3. The bacterial culture was grown to an OD_600_ of 0.1–0.3, and protein expression was induced overnight with 0.1 mM IPTG. Subsequently, the cells were lysed *via* sonication, and the protein was purified using a Ni–NTA affinity chromatography column. Purified proteins were utilized for Western blot (WB) analysis as described by Yu et al. (2020). The purified proteins were separated through SDS-PAGE electrophoresis. Following electrophoresis, the separated proteins were transferred to a PVDF membrane and blocked with 5% skimmed milk. Antibody incubation, washing, secondary antibody incubation, and detection were then conducted. Protein detection was performed *via* chemiluminescence or staining. His-tag mouse monoclonal antibody (Pearland, TX, USA) and HRP-conjugated goat anti-mouse IgG (Pearland, TX, USA) were employed for WB analysis. The WB assay utilized the EasySee® Western Marker (25–90 kDa). This marker contains protein bands with multiple IgG subtype-binding sites, enabling simultaneous antibody incubation of both molecular weight standard bands and target protein bands using the same labeled antibody. Protein bands were subsequently visualized through chemiluminescence detection. The enzyme activity assay for AcAAT4 comprised 50 µg of purified protein, 10 µL of 10 mM (*Z*)− 3-hexenol, 50 µL of acetyl-CoA, and 50 mM Tris–HCl buffer (pH 7.4) to a final volume of 1 mL. The reaction mixture was incubated at 30 °C, and the products were adsorbed using SPME for 1 h. The samples were analyzed using a gas chromatography − mass spectrometer (GC − MS) QP2010 SE (Shimadzu Corporation, Kyoto, Japan) equipped with a GC column (SUPELCOWAX- 10, 30 m × 0.25 mm × 0.25 μm, Supelco Inc., Bellefonte, PA, U.S.A.). The injector temperature was maintained at 240 °C for 1 min. The helium (carrier gas) flow rate was set at 1.0 mL/min. The initial column temperature was held at 60 °C for 3 min, followed by a temperature increase to 240 °C at a rate of 4 °C/min, and maintained at 240 °C for 20 min. Mass spectrometry (Shimadzu Corporation, Kyoto, Japan) was operated in full scan mode (mass range, *m/z* 40–200).

### Transient expression in *N. benthamiana*

The recombinant plasmid AcAAT4-GFP was constructed by ligating the AcAAT4 to the expression vector pEAQ-GFP. The relevant primers are presented in Table S3. Subsequently, AcAAT4-GFP was transformed into *Agrobacterium tumefaciens GV3101*. When the culture turned orange, the cells were centrifuged and resuspended in infiltration medium to an OD_600_ of 0.5. The suspension was injected into tobacco leaves, with pEAQ-GFP empty vector serving as a control. The plants were incubated at 25 °C under normal photoperiod for 72 h. Following incubation, the tobacco leaves were cut and placed in 500 μL of 0.5 M (*Z*)− 3-hexenol solution for 2 h. After complete absorption, the leaves were further cultured in water, and the aroma compounds were collected using SPME for 30 min. Samples were analyzed by GC–MS using the instrument parameters as described in Sect. 2.10.

### Transient transcription activation assay in *N. benthamiana*

The *AcMYC2b* sequence was inserted into the pEAQ-GFP vector to construct the effector *AcMYC2b-GFP*, with the empty pEAQ-GFP vector serving as a control. A 1500 bp fragment from the AcAAT4 promoter region was cloned into the pGreenII 0800-LUC vector to construct the reporter gene (AcAAT4 reporter). The Renilla luciferase (REN) gene in the vector was utilized as an internal reference. The recombinant plasmids AcMYC2b-GFP, AcAAT4 reporter, and empty pEAQ-GFP were transferred into *GV3101*. Subsequently, these *GV3101* were infiltrated into tobacco leaves for transient expression. Each treatment comprised 5 replicates, with one tobacco plant per replicate. (1) After 48 h of cultivation, 100 μM luciferin was applied to the abaxial surface of tobacco leaves and incubated in darkness for 5 min. Images were then captured using a low-light cooled charge-coupled device (CCD) imaging system Tanon 5200 (Tanon Science & Technology Co., Ltd., Shanghai, China) to measure fluorescence signal intensity. (2) Following gene expression, infiltrated tobacco leaves were collected, and protein was extracted. Luciferase activity was measured using a dual luciferase reporter assay system kit. The expression level of the reporter gene was indicated by the ratio of LUC to REN.

### Electrophoretic mobility shift assay

The *AcMYC2b* sequence was inserted into the pGEX4 T- 3-GST vector to construct the effector *AcMYC2b-GST*, with the empty pGEX4 T-3-GST vector serving as a control. The relevant primers are shown in Table S3. The methodology was performed as described by Han et al. (2016). The Thermo EMSA kit was utilized according to the manufacturer's instructions. Protein was combined with a digoxigenin-labeled probe to form a probe-protein complex, which was subsequently subjected to SDS-PAGE gel electrophoresis. Following electrophoresis, the gel was transferred to a membrane and cross-linked under UV light. The probe was then detected using chemiluminescence.

### Subcellular localization analysis

The target genes AcAAT4 and AcMYC2b were inserted into the pSAT6-EYFP-N1 vector. *A. thaliana* protoplasts were isolated and subsequently transformed with the recombinant vectors using the PEG-mediated transformation method. The transformed protoplasts were incubated in darkness for 12 h, after which EYFP fluorescence was observed *via* confocal microscopy to determine the subcellular localization of the target proteins. The specific method followed the protocol reported by Jian et al. (2021).

### Statistical analysis

Data analysis and determination of differences between two groups were conducted using *t*-tests in Excel and SPSS 18.0 software (SPSS Inc.). For comparisons involving three or more groups, one-way analysis of variance (ANOVA) and Duncan's multiple comparison test were performed using SPSS software (Ver. 25.0, SPSS Inc., Chicago, IL, USA).

## Supplementary Information

Additional file 1: Fig. S1. Atmospheric conditions of field experiment with slow-release agents in 2024. Fig. S2. Analysis of the effects of (*Z*)- 3-hexenyl acetate on insect behavior from 29 May to 18 June, 2024. Fig. S3. Visualization analysis of the degree of damage in tea leaves. Fig. S4. Evaluation of the degree of damage to tea leaves under *Empoasca onukii* Matsuda treatment. Fig. S5. Transcriptomic analysis of tea leaves fumigated with (*Z*)- 3-hexenyl acetate. Fig. S6. Metabolomic analysis of tea leaves fumigated with (*Z*)- 3-hexenyl acetate. Fig. S7. Analysis of in vitro enzymatic activities of AcAATs. Fig. S8. GC–MS results of transiently expressed AcAAT4 in tobacco. Fig. S9. Phylogenetic analysis of the AcMYC2 s transcription factor. Fig. S10. Gene suppression of *AcAAT4* in *Acacia confusa*. Table S1. Analysis of the main volatiles of *Acacia confusa* Merr.. Table S2. Primer pairs used for qPCR. Table S3. Primer sequence used for gene amplification.

## Data Availability

All pertinent data are included within the manuscript and accompanying supplementary information files.

## Abbreviations

AAT: Alcohol acyltransferase
MYC2: Myelocytomatosis protein 2
EMSA: Electrophoretic mobility shift assays
GFP: Green fluorescent protein
LUC: Luciferase
REN: Renilla luciferase
PEG: Polyethylene glycol
EYFP: Enhanced yellow fluorescent protein​
SDS-PAGE: Sodium dodecyl sulfate–polyacrylamide gel electrophoresis
PVDF: Polyvinylidene fluoride membrane
JA: Jasmonic acid
