# Supplementary material for: Mechanism mediating the biosynthesis of the anti-insect volatile (Z)-3-hexenyl acetate in Acacia confusa Merr., an intercropping plant in tea plantations
Source: Mol Hortic. 2025 Aug 7;5:47. doi: 10.1186/s43897-025-00165-z (PMC12330005; doi:10.1186/s43897-025-00165-z)
Supplement: Supplementary file 1 — Additional file 1: Fig. S1. Atmospheric conditions of field experiment with slow-release agents in 2024. Fig. S2. Analysis of the effects of (Z)- 3-hexenyl acetate on insect behavior from 29 May to 18 June, 2024. Fig. S3. Visualization analysis of the degree of damage in tea leaves. Fig. S4. Evaluation of the degree of damage to tea leaves under Empoasca onukii Matsuda treatment. Fig. S5. Transcriptomic analysis of tea leaves fumigated with (Z)- 3-hexenyl acetate. Fig. S6. Metabolomic analysis of tea leaves fumigated with (Z)- 3-hexenyl acetate. Fig. S7. Analysis of in vitro enzymatic activities of AcAATs. Fig. S8. GC–MS results of transiently expressed AcAAT4 in tobacco. Fig. S9. Phylogenetic analysis of the AcMYC2 s transcription factor. Fig. S10. Gene suppression of AcAAT4 in Acacia confusa. Table S1. Analysis of the main volatiles of Acacia confusa Merr.. Table S2. Primer pairs used for qPCR. Table S3. Primer sequence used for gene amplification. [file 43897_2025_165_MOESM1_ESM.docx]

**Running title:** (*Z*)-3-Hexenyl acetate biosynthesis in *Acacia confusa* Merr.

**Title:** Mechanism mediating the biosynthesis of the anti-insect volatile (*Z*)-3-hexenyl acetate in *Acacia confusa* Merr., an intercropping plant in tea plantations

**Authors:** Guotai Jian ^1, 3, 4, #^, Jianlong Li ^2, #^, Yong Wu ^5^, Chengshun Liu ^1, 4^, Ronghua Li ^1, 4^, Jiajia Qian ^1, 3^, Yongxia Jia ^1^, Hanxiang Li ^1^, Jinchi Tang ^2^, Lanting Zeng ^1, 3, 4, *^

***Affiliation*:**

*^a^ Guangdong Provincial Key Laboratory of Applied Botany & State Key Laboratory of Plant Diversity and Specialty Crops, South China Botanical Garden, Chinese Academy of Sciences, No. 723 Xingke Road, Tianhe District, Guangzhou 510650, China*

*^b^Tea Research Institute,* *Guangdong Academy of Agricultural Sciences & Guangdong Provincial Key Laboratory of Tea Plant Resources Innovation and Utilization, No. 6 Dafeng Road, Tianhe District, Guangzhou 510640, China*

*^c^ Key Laboratory of National Forestry and Grassland Administration on Plant Conservation and Utilization in Southern China, South China Botanical Garden, Chinese Academy of Sciences, No. 723 Xingke Road, Tianhe District, Guangzhou 510650, China ^d^ University of Chinese Academy of Sciences, No.19A Yuquan Road, Beijing 100049, China*

*^5^ Yingde City Meteorological Bureau, No. 44 East Education Road, Yingde, Qingyuan, 513000 China*

**^*^ Corresponding author:** Lanting Zeng, South China Botanical Garden, Chinese Academy of Sciences, No. 723 Xingke Road, Tianhe District, Guangzhou 510650, China; Tel: +86-020-37021938; Email address: zenglanting@scbg.ac.cn.

^#^ Co-first authors.

**Supplementary Material**

**Materials and methods**

**1. The codes used in the** **Method for Quantifying Leaf Damage in Tea Leaves**

**Environment Configuration: Windows11; Matlab2020a**

```matlab

%% Clear all variables

clc;

close all;

clear all;

%% Visualize a sample from CK

Fig.(1)

img = imread('E:\Leaf Data Analysis\Image Feature Extraction\CK\1\15.tif');

imgr = img(:,:,1);

imgg = img(:,:,2);

imgb = img(:,:,3);

subplot(221);

imshow(imgr);

title('r')

subplot(222);

imshow(imgg);

title('g')

subplot(223);

imshow(imgb);

title('b')

subplot(224);

imshow(img)

title('Original Image')

%% Visualize a sample from T1

Fig.(2)

img = imread('E:\Leaf Data Analysis\Image Feature Extraction\T1\1\15.tif');

imgr = img(:,:,1);

imgg = img(:,:,2);

imgb = img(:,:,3);

subplot(221);

imshow(imgr);

title('r')

subplot(222);

imshow(imgg);

title('g')

subplot(223);

imshow(imgb);

title('b')

subplot(224);

imshow(img)

title('Original Image')

%% Visualize a sample from T2

Fig.(3)

img = imread('E:\Leaf Data Analysis\Image Feature Extraction\T2\1\15.tif');

imgr = img(:,:,1);

imgg = img(:,:,2);

imgb = img(:,:,3);

subplot(221);

imshow(imgr);

title('r')

subplot(222);

imshow(imgg);

title('g')

subplot(223);

imshow(imgb);

title('b')

subplot(224);

imshow(img)

title('Original Image')

%% Visualize a sample from T3

Fig.(4)

img = imread('E:\Leaf Data Analysis\Image Feature Extraction\T3\1\15.tif');

imgr = img(:,:,1);

imgg = img(:,:,2);

imgb = img(:,:,3);

subplot(221);

imshow(imgr);

title('r')

subplot(222);

imshow(imgg);

title('g')

subplot(223);

imshow(imgb);

title('b')

subplot(224);

imshow(img)

title('Original Image')

%% Read

for i = 1:20 %1-20 subfolders

for j = 1:20 %1-20 tif format RGB image files in each subfolder

filename = strcat('E:\Leaf Data Analysis\Image Feature Extraction\T3\',num2str(i),'\',num2str(j),'.tif'); %Read "E:\Leaf Data Analysis\Image Feature Extraction\CK" 1-20 files each with 1-20 .tif images

A = imread( filename ); %Read all RGB images

A1= rgb2gray(A); %Convert RGB image A to grayscale image A1

r = A(:,:,1);

g = A(:,:,2);

b = A(:,:,3);

%% Calculate the mean and variance of color features

R=mean2(r);%Mean RGB model

G=mean2(g);

B=mean2(b);

sR=std2(r);%Variance RGB model

sG=std2(g);

sB=std2(b);

%% Create an empty matrix

T1=[];

T=[R; G; B; sR; sG; sB];%Statistical feature matrix

T=T;

T1=[T1;T];%Sample set, each sample's features as a column

I{i,j} = T1;

end

end

%% Read data from T{i,j} to form a data matrix

C1=[];

for i=1:20

Z=[I{i,1} I{i,2} I{i,3} I{i,4} I{i,5} I{i,6} I{i,7} I{i,8} I{i,9} I{i,10} I{i,11} I{i,12} I{i,13} I{i,14} I{i,15} I{i,16} I{i,17} I{i,18} I{i,19} I{i,20} ];%Extract data from the 1-20 columns of row i in the I database to form Z

Z=Z';

C1=[C1;Z];

end

%% Calculate the average of all samples for this treatment

C2=mean(C1)';

%% Save the results to an Excel file

writematrix(C2, 'T3.xlsx');% Change the save file name according to the imported treatment

%% Output 20*20 data

writematrix(C1, 'All images' features of T3.xlsx');% Change the save file name according to the imported treatment

**2. Gene suppression of *AcAAT4* in *Acacia confusa* Merr.**

The functional assay of AcAAT4 was carried out by gene suppression of AcAAT4 in *Acacia confusa* Merr.. The method was adapted from the previously reported paper (Jin et al., 2025). Candidate sequences of the antisense oligonucleotides (AsODNs) with complementarity to the segment of AcAAT4 were selected using Soligo software (http://sfold.wadsworth.org/cgi-bin/index.pl) with AcAAT4 sequences as inputs. Three primers were designed and synthesized by Beijing Tsingke Biotech Co., Ltd. (Beijing, China). The sequences were as follows: ​AsODN-AcAAT4: TAGTTCTGGTTGGCGTCTCC, TTAGTTCTGGTTGGCGTCTC, and ATTAGTTCTGGTTGGCGTCT; ​sODN-AcAAT4: GGAGACGCCAACCAGAACTA, GAGACGCCAACCAGAACTAA, and AGACGCCAACCAGAACTAAT. Naturally growing tender *Acacia confusa* Merr. shoots with five-six leaves were excised and placed in Eppendorf tubes containing 1 mL of 50 µM AsODN-AcAAT4 (to suppress AcAAT4) for 24 h, the sense oligonucleotides were used as control, the ddH_2_O was used as blank control. Five replicates were conducted for the treatment and control. All leaves were harvested and kept at -80 ^o^C prior to analysis. The solid-phase microextraction (SPME) (2 cm-50/30 μm DVB/Carboxen/PDMS Stable Flex, Supelco Inc., Bellefonte, PA, USA) was placed into the head space of the 250 mL beaker to collect volatiles for 30 min. Afterwards, the volatiles collected by the SPME were analyzed by gas chromatography–mass spectrometry (GC–MS). The GC–MS system was equipped with a SUPELCOWAX 10 column (30 m×0.25 mm×0.25 μm, Supelco Inc., Bellefonte, PA, USA). The injector temperature was 240 °C for 1 min. The helium (carrier gas) flow rate was 1.0 mL/min. The initial column temperature was 60 °C for 3 min, then the initial GC oven temperature was 40 °C for 3 min, which was then ramped to 140 °C at a rate of 2 °C/min and ramped to 240 °C at a rate of 20 °C/min, and held at 240 °C for 20 min. Mass spectrometry (Shimadzu Corporation, Kyoto, Japan) was operated in full scan mode (mass range, *m/z* 40–200).

**3. Transcript expression analysis of genes**

Gene transcript expression was determined by quantitative real-time polymerase chain reaction (qRT-PCR). Total RNA was obtained using Quick RNA isolation Kit (Huayueyang Bio., Co., Ltd., Beijing, China) from tea leaves. The cDNA was reversely transcribed from total RNA using PrimeScript^TM^ RT reagent Kit (Takara Bio Inc., Kyoto, Japan.) according to the manufacture’s instruction. RNA (1 μg) was pipetted for reverse transcription, and then diluted 20-fold. The primers were designed and synthesized by Beijing Tsingke Biotech Co., Ltd. (Beijing, China). The sequences were as follows: forward primer, ACGGCGGAGTATATGCAATC; reverse primer, CTCCTTTTGCACTCCCACAT. The qRT-PCR reactions system consisted of 5 μL of iTaq^TM^ Universal SYBR^®^ Green Supermix (Bio-Rad Laboratories, Hercules, CA, USA), 2.5 μL template cDNA, 2.5 μL of each forward and reverse primers (primer reservoir solutions concentration is 10 μM, diluted 10 times) and sterile water in a 10 μL total volume. *Encoding elongation factor 1* (*AcEF1-α*) were used as an internal reference gene. The qRT-PCR was carried out on Roche LightCycle 480 (Roche Applied Science, Mannheim, Germany) under condition of one cycle of 95 °C for 60 s, 40 cycles of 95 °C for 15 s, and 60 °C for 60 s. A melt curve was performed at the end of each reaction to verify PCR product specificity. The 2^-△△ct^ method was used to calculate the relative expression level. Changes in mRNA levels of related genes were normalized to that of *AcEF1-α*.

**4. Sequence of *Alcohol Acyltransferase 4* (*AcAAT4*)**

ATGGCTTCACCTTCAGCATTTTCCAGTGATGTAGTGTTTGCTGTGAGGAGGCGCCAACCAGAGCTGGTGGCTCCGGCGAAGCCCACCCCTCATGAACTGAAACTTCTTTCGGACATAGATGACCAACAAGGCTTACGATTTCAGATACCAATGATACATTTTTATGGTCACAAGGTATCCATGCAAGGAAAGGACCCAGCTCAAGTCATCAAGAAGGCTCTGTCTCAAGCTTTAGTGTTTTATTACCCATTTGCAGGTAGACTCAAGGAAGGGCCTGGACGCAAGCTTATGGTTGACTGTAATGAAGAAGGAGTCATCTTCATCGAAGCCGATGCCGATATCACTCTTCAACAATTTGGTAGCATTCTTCAACCACCATTCCCATATTTTGATGAACTTCTTTATAAAGTTCCTGGTTCTGAAGGAGTTATCAACTGTCCCCTTTTAATTATCCAGGTTACACGCCTCAAGTGTGGTGGATTCATCTTTGCCTACCGCCTAAATCACACTATGAGTGATGGTGCTGGCATAGTCCAATTCCTAAACGCCGTGGCTGAGATAGCCGGCGGAGCATCTGAACCTTCGATCGCTCCGGTATGGCAGAGAGAGCTTTTATCAGCAAGAGACCCGCCTCGAATTACTTGCACCCATCGGGAGTTCGAGCAAGTGCCTAATAATTATGATCCAATATGGACACAACAAAGCATCACTCACCAATCTTTCTTCTTTGGACCCAAAAAGCTAGAGGCCATCCGTCAATTGTTCTCCCATTATAATGCTCACTACGTTACTAGGTTTGAAGCTCTCACAGCTTTCCTATGGCGTTGTCGTACTAAAGCATTGCAATTAGAACCCTACGAGGAGGTTCGCTTTTTTTGTATCAATAATATAAGGGGCAAATCAAATCCTCCATTATTGCCACTTGGTTATTATGGCAATTCTTTTGCGTACCCAGCTGCAGTCACAACCGCAGGGAAGCTCTGTGAGAATTCATTAGGGTTTGCCTTAGAGTTGGTGAAGAAGGCGAAAGCTCAGGCGACAACGGAGTATATACAATCTGTGGCTGATCTTATGGTCATTCAAGGACGACCCTGCTTCACTATGCCTGGATCATGGTTTGTGTCGGATACGTCACGTCTTGGGTTTAGAGGTGTGGATTTTGGGTGGGGTAAGGCGGTATACGGTGGTCCGGCAACAGGAGGAGCTGGTCCCTTTCCCGGAGTAAGCTATTTTGTTGCATGTGAGAATGCAAAACGAGAGGAAGGAATAGTTGTGCCATTCTACTTGCCGCTCAATGCTATGAAGAGGTTCGTTGAGGAATTGGATCAAGTACTTGGGCAACTTTAA

Table S1 Analysis of the main volatiles of *Acacia confusa* Merr.

| No. | CAS | Volatile compound | RI  (Calculated) | RI  (Referred) | Qualitative method |
| --- | --- | --- | --- | --- | --- |
| 1 | 3208-16-0 | 2-Ethylfuran | 933 | 936  (Kawakami et al, 1991) | RI, MS |
| 2 | 143-22-6 | Butoxytriglycol | 959 | - | MS |
| 3 | 108-88-3 | Toluene | 1050 | 1049  (Ciążyńska et al, 2003) | RI, MS |
| 4 | 13466-78-9 | 3-Carene | 1126 | 1127  (Bicchi et al, 2003) | RI, MS |
| 5 | 106-42-3 | *p*-Xylene | 1133 | 1137  (Chung et al, 2002) | RI, MS |
| 6 | 108-38-3 | Benzene, 1,3-dimethyl- | 1134 | 1136  (Cha et al, 1998) | RI, MS |
| 7 | 100-41-4 | Ethylbenzene | 1152 | 1158  (Vernon et al, 1983) | RI, MS |
| 8 | 42125-10-0 | (*Z*)-2-Penten-1-ol acetate | 1184 | - | MS |
| 9 | 17957-93-6‌ | Geranyl vinyl ether | 1214 | - | MS |
| 10 | 5989-27-5 | D-Limonene | 1233 | 1230  (Tu et al, 2002) | RI, MS, Std |
| 11 | 3681-71-8 | (*Z*)-3-Hexen-1-ol acetate | 1315 | 1314  (Iwaoka et al, 1994) | RI, MS, Std |
| 12 | 111-27-3 | 1-Hexanol | 1342 | 1348  (Zhu et al, 2015) | RI, MS, Std |
| 13 | 928-96-1 | (*Z*)-3-Hexen-1-ol | 1420 | 1442  (Beauchêne et al, 2000) | RI, MS, Std |
| 14 | 119-36-8 | Methyl salicylate | 1725 | 1727  (Yamaguchi et al, 1981) | RI, MS, Std |
| 15 | 91-20-3 | Naphthalene | 1726 | 1728  (Kanasawud et al, 1990) | RI, MS |

No., Number; RI, retention index; MS, mass spectrometry; Std, standard.

Table S2 Primer pairs used for qPCR

| Gene | Forward primer 5'-3' | Reverse primer 5'-3' |
| --- | --- | --- |
| *AcEF1-α* | TTCCAAGGATGGGCAGAC | TGGGACGAAGGGGATTTT |
| *AcAAT1* | CCACGGGCTATTTCACCTAA | ATCAGATCTGTCGCGTGTTG |
| *AcAAT2* | CACAACTCTGCCCTCCTCTC | GTACCCAGCTGCAGTCACAA |
| *AcAAT3* | CGACACAAACCATGATCCAG | ACGGCGGAGTATATGCAATC |
| *AcAAT4* | ACGGCGGAGTATATGCAATC | CTCCTTTTGCACTCCCACAT |
| *AcMYC2a* | AAAAACGACCTCGCAAGAGA | CAGGGATGCTTTGTCCATTT |
| *AcMYC2b* | TTTGGACCGACGATAACTCC | GGAGACGTTGCTGGAGAGTC |

*AcEF1-α*, *encoding elongation factor 1*; *AcAAT*, *alcohol acyl transferase*; *AcMYC2*, *myelocytomatosis protein 2*.

Table S3 Primer sequence used for gene amplification

| Gene name | Forward primer 5'-3' | Reverse primer 5'-3' |
| --- | --- | --- |
| *AcAAT1-his* | gacaaggccatggctgatatcATGGCCCCATCAGCATTATCA | ttgtcgacggagctcgaattcTAATGGAGATGTGATAAATAACCTCGG |
| *AcAAT2-his* | gacaaggccatggctgatatcATGGTTTTACTGACTTCCTCTCATCTC | ttgtcgacggagctcgaattcCAAGTGAGACATCATAATCCTGGAA |
| *AcAAT3-his* | gacaaggccatggctgatatcATGGCTTCACCTTCAGCATTTT | ttgtcgacggagctcgaattcAAGTTGCCCAAGTACTTGATCCA |
| *AcAAT4-his* | gacaaggccatggctgatatcATGGCTCAACCATCTACTTTGGA | ttgtcgacggagctcgaattcTAGACAAGACTTGATAAATTTAGAATATTGACC |
| *AcAAT4-GFP* | caaattcgcgaccggtATGGCTCAACCATCTACTTTGGA | tgctagtcataccggtTAGACAAGACTTGATAAATTTAGAATATTGACC |
| *AcAAT4-YFP* | cgacggtaccgcgggcccgggATGGCTCAACCATCTACTTTGGA | gctcaccatcaggatcccgggTAGACAAGACTTGATAAATTTAGAATATTGACC |
| *AcMYC2b-His* | gacaaggccatggctgatatcATGAATCTTTGGACCGACGAT | ttgtcgacggagctcgaattcTCGGACATCGCCAACTTTGGA |
| *AcMYC2b-YFP* | cgacggtaccgcgggcccgggATGAATCTTTGGACCGACGAT | gctcaccatcaggatcccgggTCGGACATCGCCAACTTTGGA |
| *AcMYC2b-GFP* | CAAATTCGCGACCGGTATGAATCTTTGGACCGACGAT | TGCTAGTCATACCGGTTCGGACATCGCCAACTTTGGA |
| *AcMYC2b-GST* | GTGGATCCCCGAATTCCATGAATCTTTGGACCGACGAT | AGTCGACCCGGGAATTCTCGGACATCGCCAACTTTGGA |

*AcEF1-α*, *encoding elongation factor 1*; *AcAAT*, *alcohol acyl transferase*; *AcMYC2*, *myelocytomatosis protein 2*.


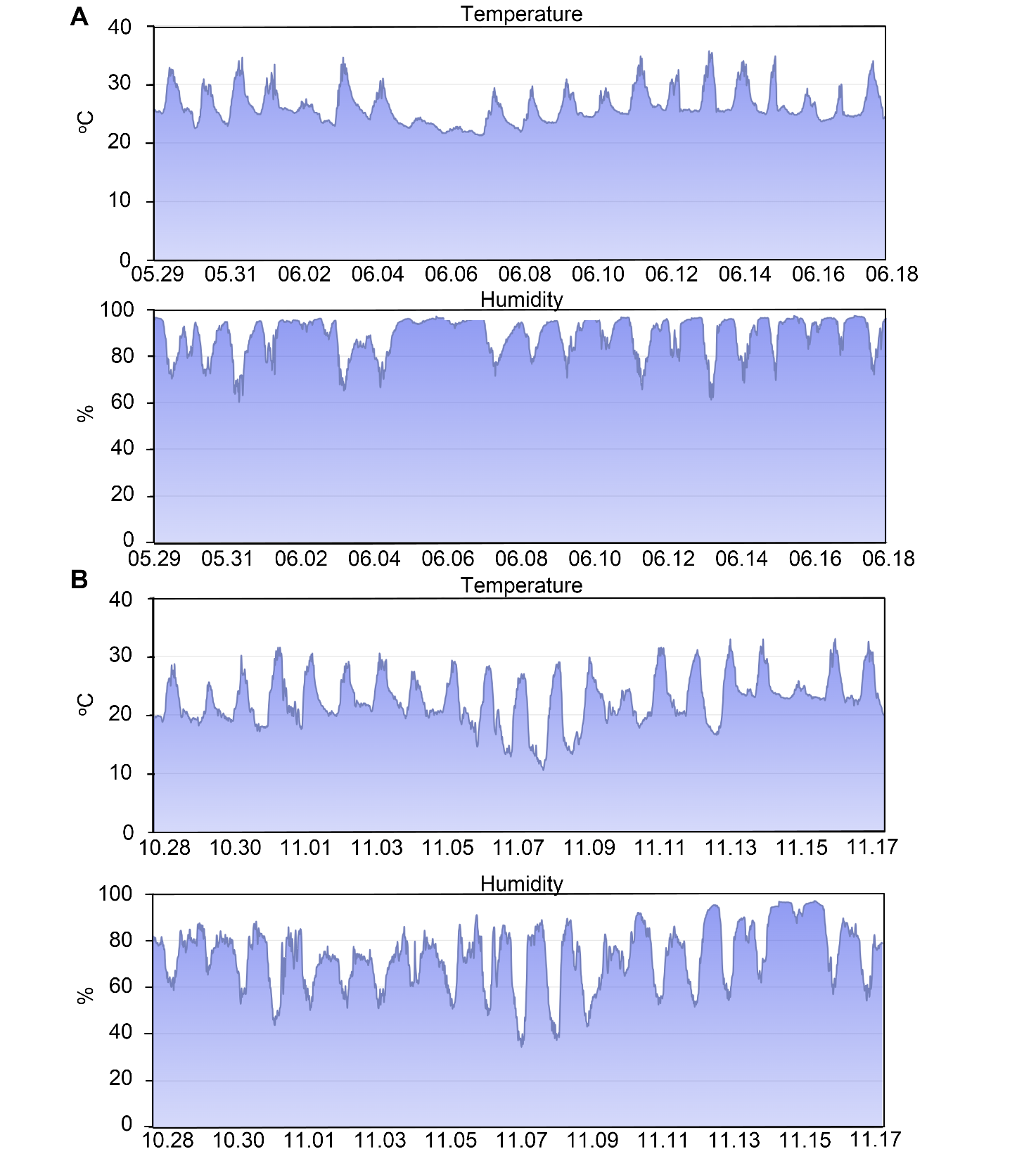


**Fig. S1 Atmospheric conditions of field experiment with slow-release agents in 2024.**


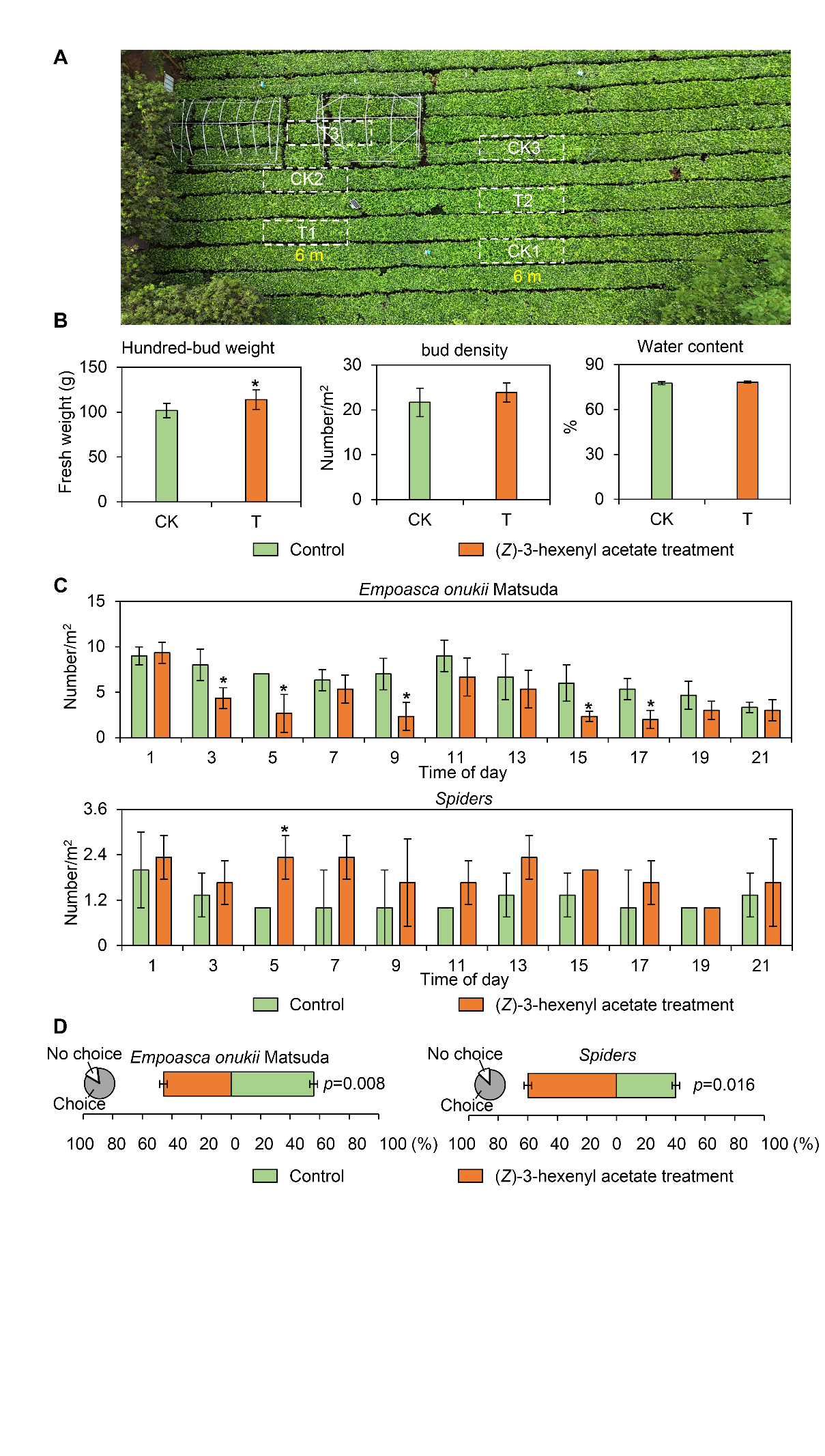


**Fig. S2 Analysis of the effects of (*Z*)-3-hexenyl acetate on insect behavior from 29 May to 18 June, 2024.**

(A) Field layout of (*Z*)-3-hexenyl acetate slow-release agents with three replicates, each replicate measuring 1.2 m × 6 m. (B) Statistical analysis of tea yield indicators 14 days after the (*Z*)-3-hexenyl acetate slow-release agents’ treatment. (C) Effect of (*Z*)-3-hexenyl acetate slow-release agents on *Empoasca onukii* Matsuda and its predatory spider. (D) Laboratory selection assay of (*Z*)-3-hexenyl acetate. Each group consisted of at least 30 replicates, with each insect serving as a replicate. Data are presented as the mean ± standard deviation (n ≥ 30). Significant differences between control and treatment groups are indicated (*, *p* ≤ 0.05 and **, *p* ≤ 0.01).


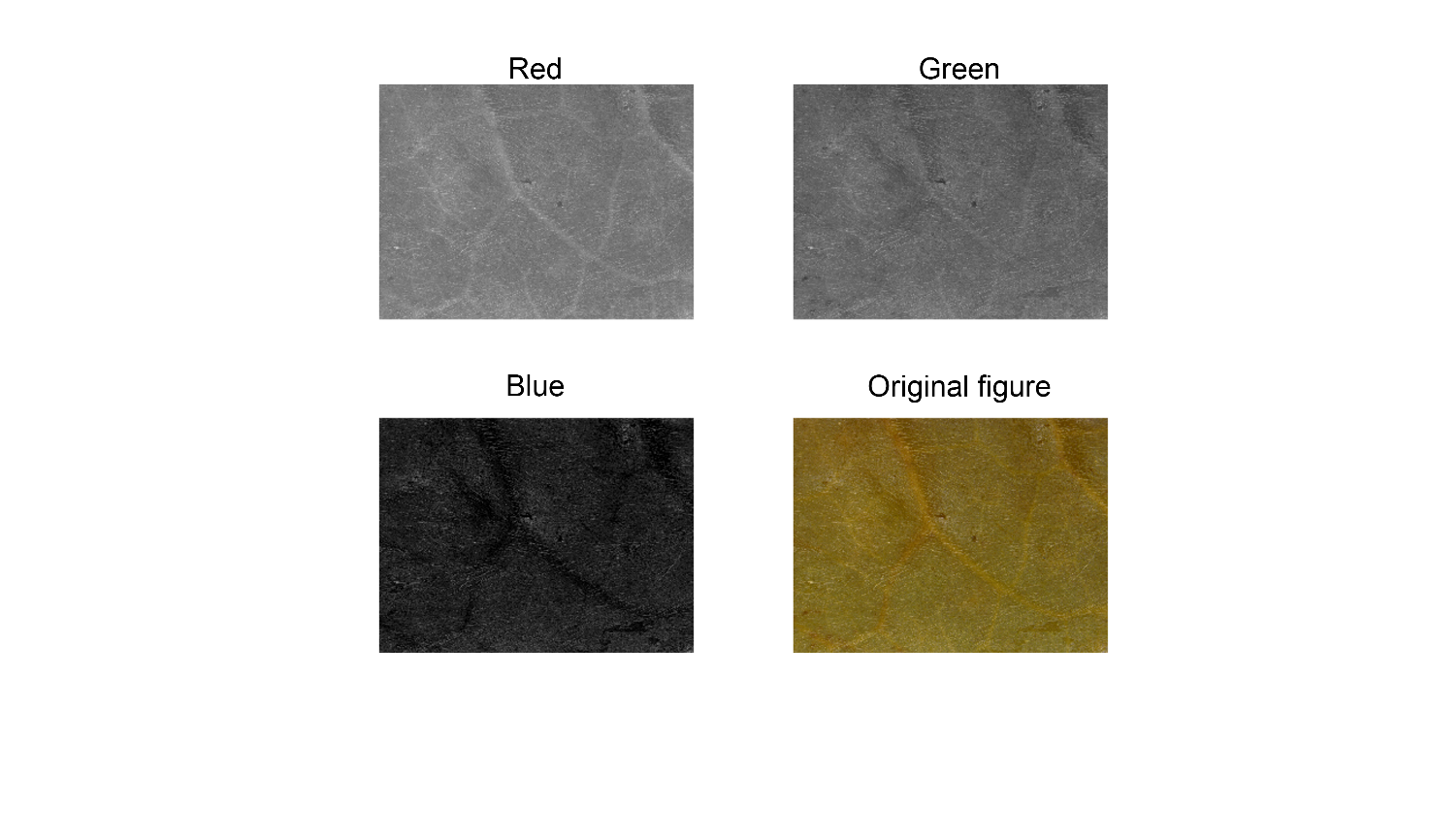


**Fig. S3 Visualization analysis of the degree of damage in tea leaves.**

The red, green and blue represent the three colour channels of RGB, which were extracted from the damaged leaves by the program. The degree of leaf damage was represented by the ratio of Red/Green. A ratio greater than 1 indicated a redder color, indicating a higher degree of damage to the tea leaves.


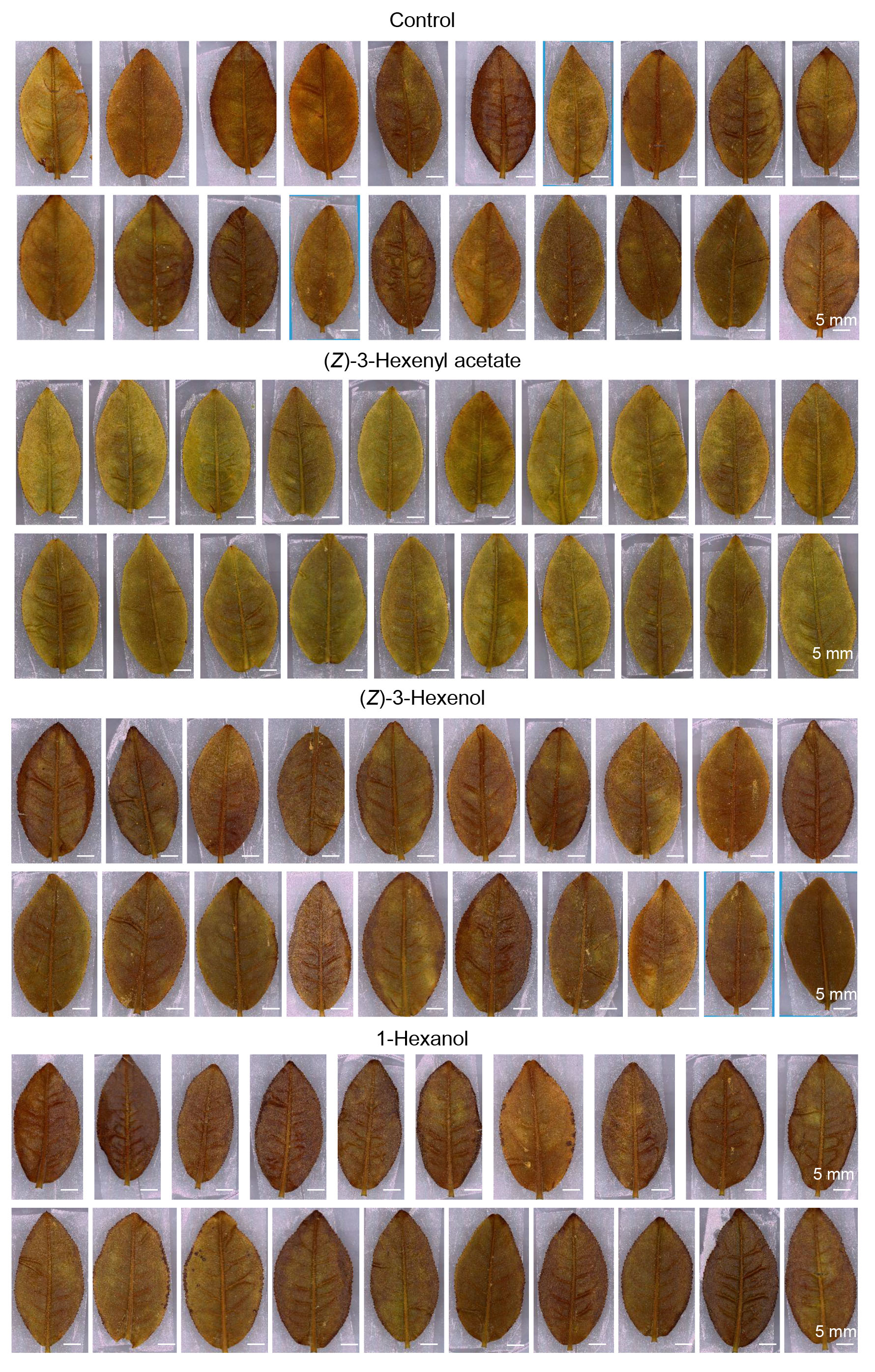


**Fig. S4 Evaluation of the degree of damage to tea leaves under *Empoasca onukii* Matsuda treatment.**


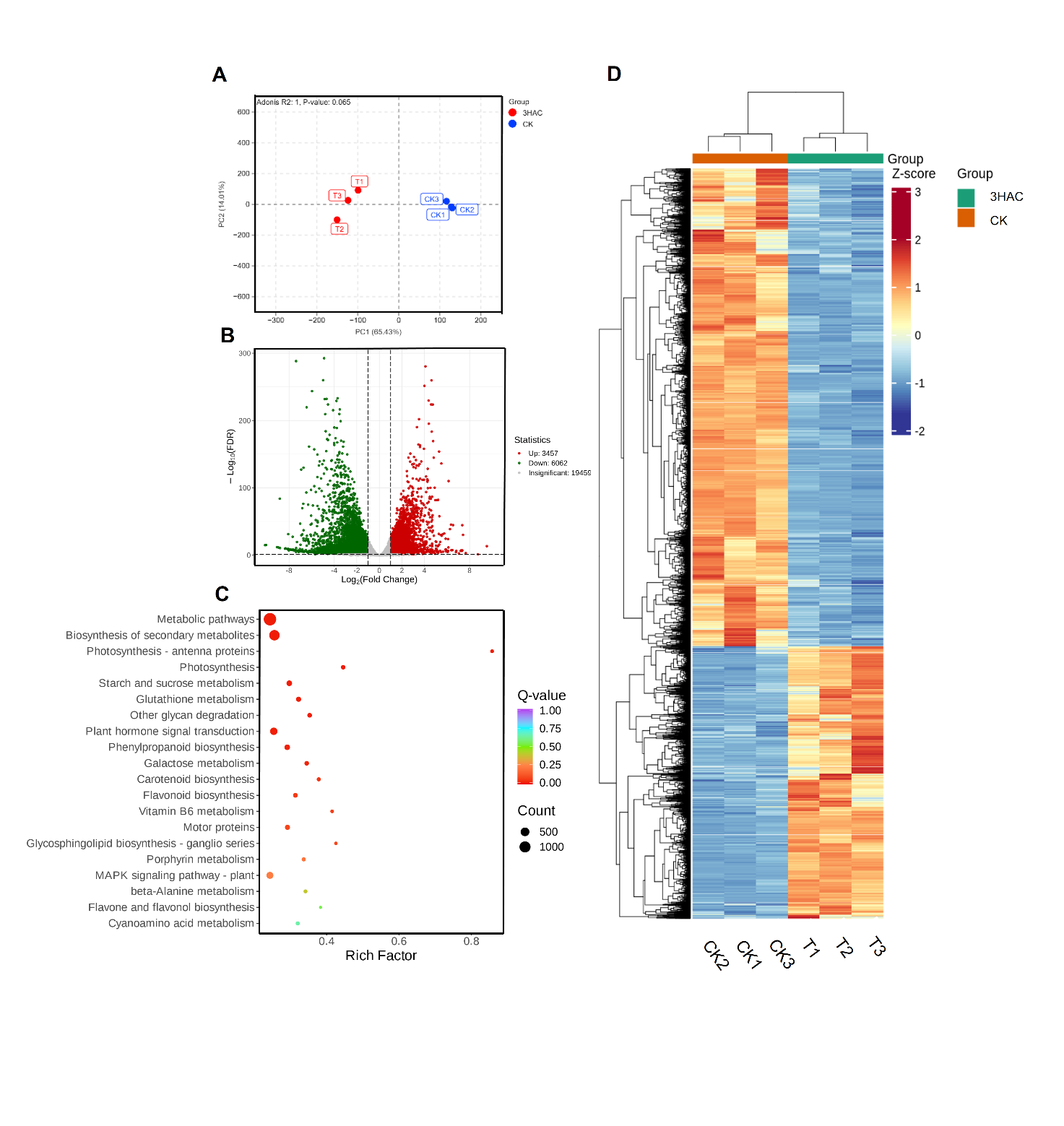


**Fig. S5 Transcriptomic analysis of tea leaves fumigated with (*Z*)-3-hexenyl acetate.**

(A) Principal component analysis (PCA), (B) volcano plot, (C) bubble chart of KEGG pathways, (D) heat map of DEGs in tender tea leaves fumigated with (*Z*)-3-hexenyl acetate solutions (prepared in dichloromethane) and dichloromethane.

**
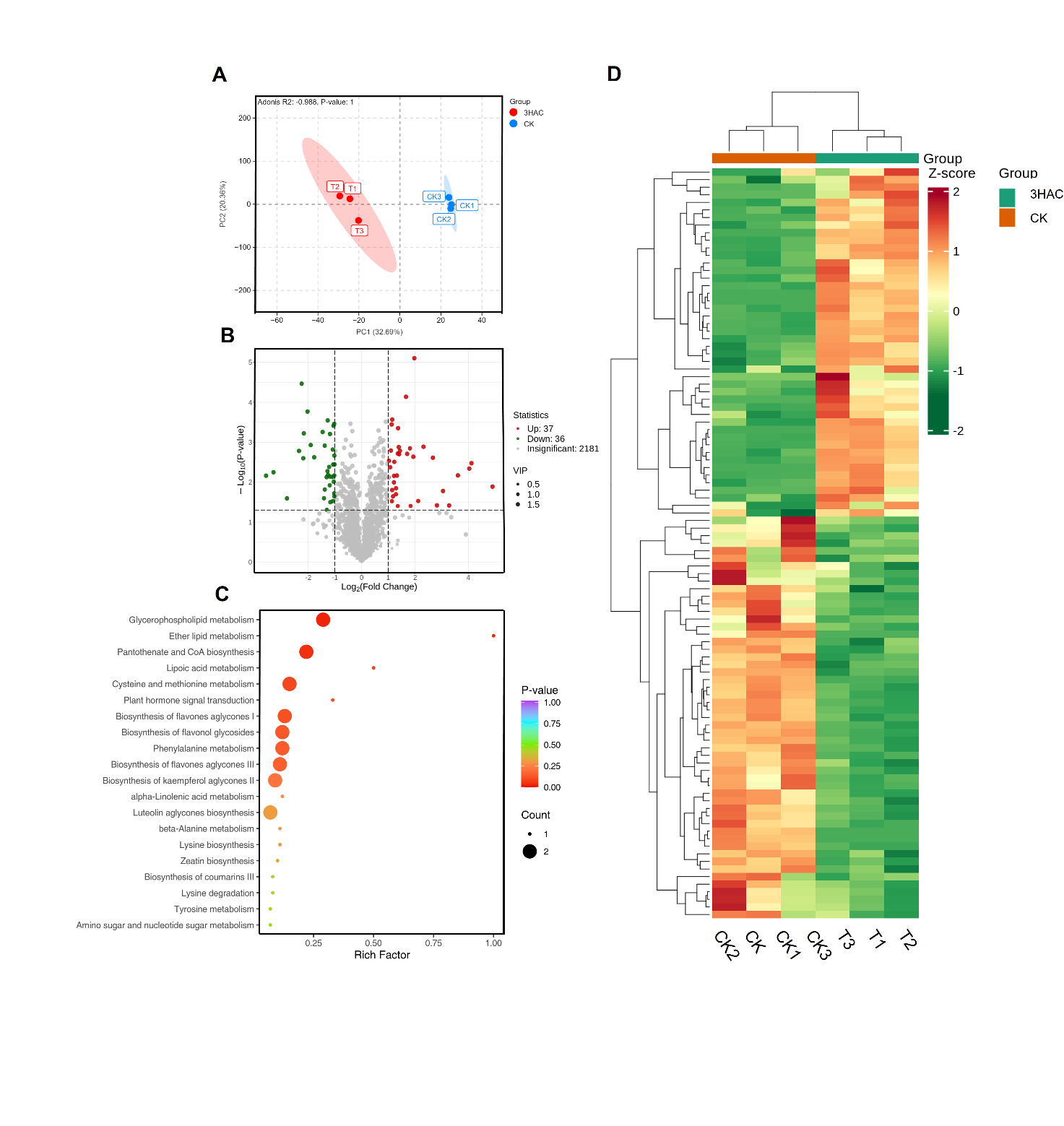
**

**Fig. S6 Metabolomic analysis of tea leaves fumigated with (*Z*)-3-hexenyl acetate.**

(A) Principal component analysis (PCA), (B) volcano plot, (C) bubble chart of KEGG pathways, (D) heat map of DEGs in tender tea leaves fumigated with (*Z*)-3-hexenyl acetate solutions (prepared in dichloromethane) and dichloromethane.


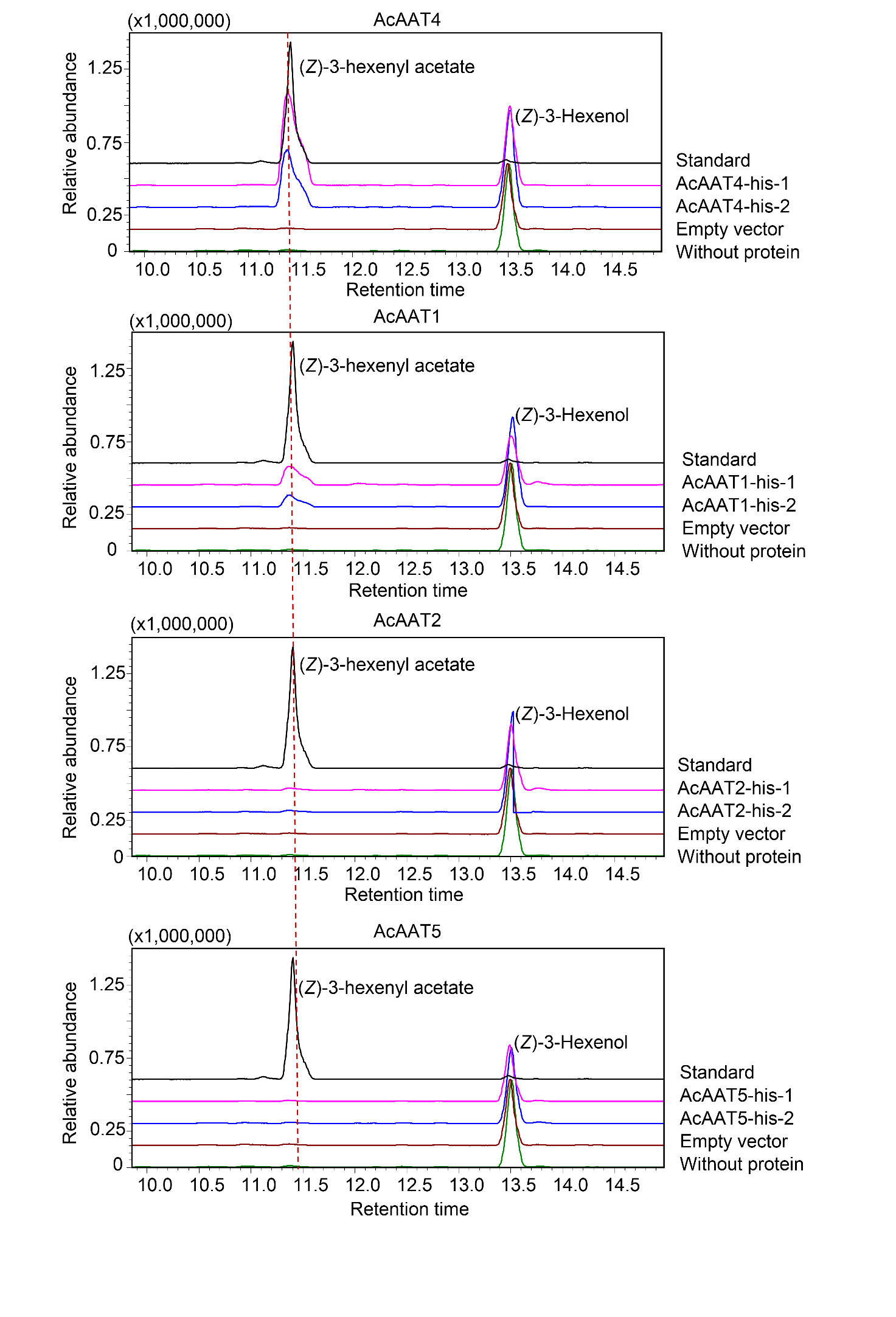


**Fig. S7 Analysis of *in vitro* enzymatic activities of AcAATs.**

*AcAAT*, alcohol acyltransferase gene.


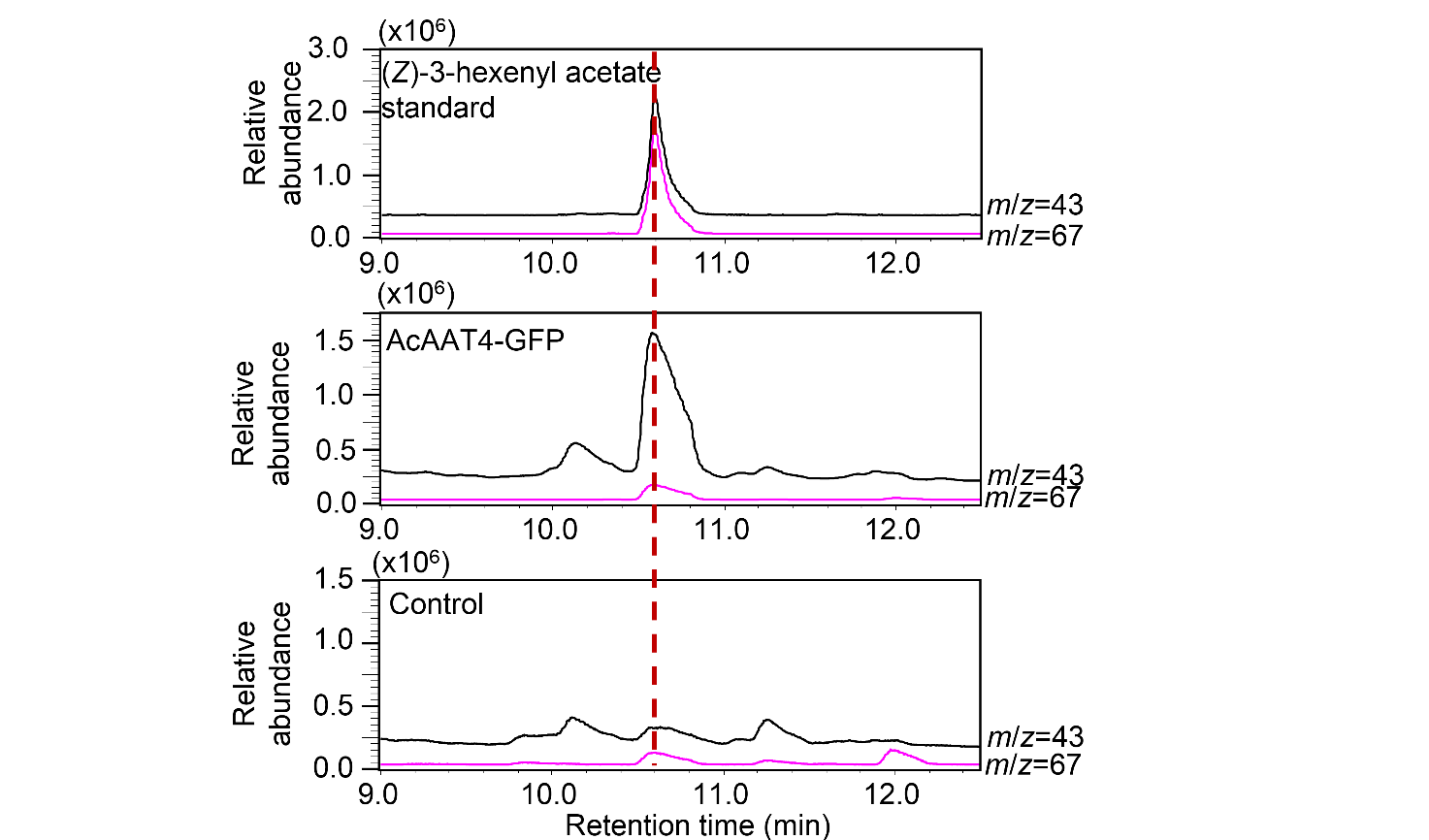


**Fig. S8 GC-MS results of transiently expressed AcAAT4 in tobacco.**

*AcAAT*, alcohol acyltransferase gene.


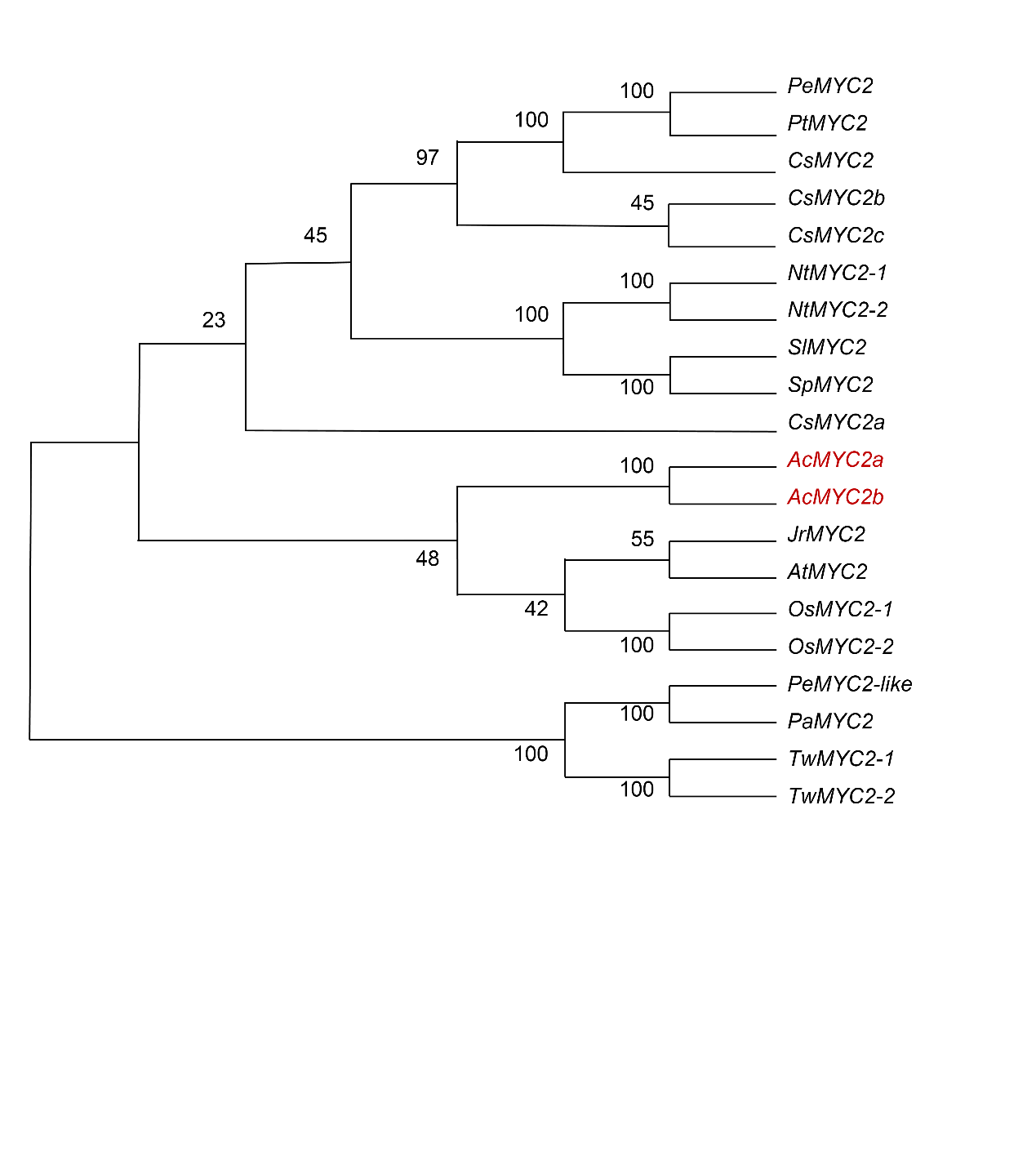


**Fig. S9 Phylogenetic analysis of the AcMYC2s transcription factor.**

*CsMYC2a/b/c: Camellia sinensis; AtMYC2: Arabidopsis thaliana; JrMYC2: Juglans regia; OsMYC2-1/2: Oryza sativa; CsMYC2: Citrus sinensis; NtMYC2-1/2: Nicotiana tabacum; PeMYC2: Populus euphratica; PeMYC2-like: Populus euphratica; PtMYC2: Populus trichocarpa; PaMYC2: Populus alba; SlMYC2: Olanum lycopersicum; SpMYC2: Solanum pennellii; TwMYC2-1/2: Tripterygium wilfordii. AcMYC2a/b: A. confuse*; *MYC2*, *myelocytomatosis protein 2*.

**
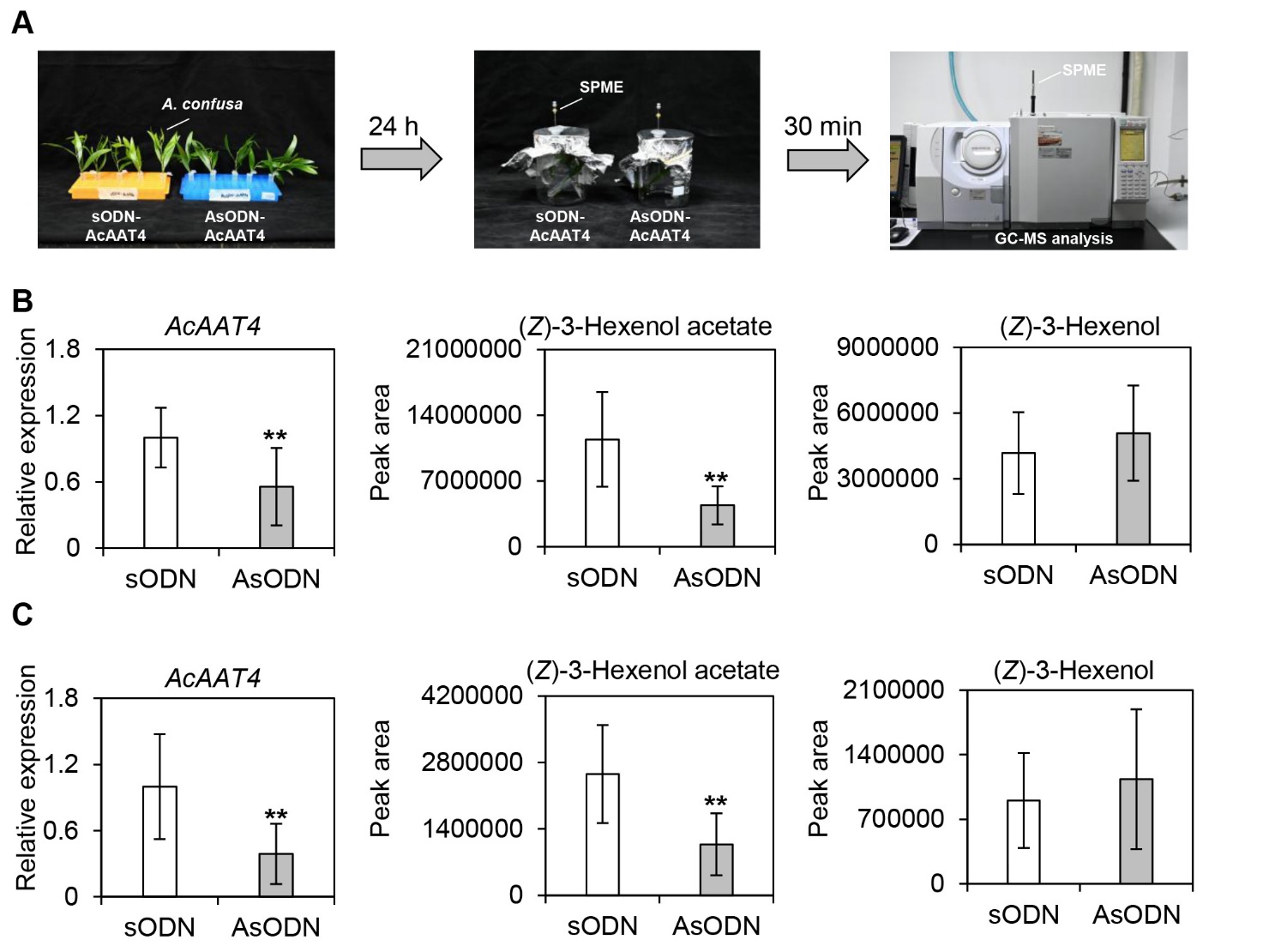
**

**Fig. S10 Gene suppression of *AcAAT4* in *Acacia confusa* Merr..**

(A) Schematic diagram of the antisense oligonucleotide experimental workflow. (B-C) Experimental results from two independent biological replicates. Data are expressed as mean±SD (n = 5). **, *p*<0.01, comparison between control and AsODN-AcAAT4 treatment.

**Reference**

Beauchêne D, Grua-Priol J, Lamer T, Demaimay M, Quémeneur F. Concentration by pervaporation of aroma compounds from *Fucus serratus*. Journal of Chemical Technology & Biotechnology. 2000; 75(6): 451-458.

Bicchi C, Rubiolo P, Saranz CEE, Vilegas W, de Souza Gracioso J, Monteiro SB. Components of *Turnera diffusa Willd*. var. afrodisiaca (Ward) Urb. essential oil. Flavour and fragrance Journal. 2003; 18(1): 59-61.

Cha YJ, Kim H, Cadwallader KR. Aroma-active compounds in kimchi during fermentation. Journal of Agricultural and Food Chemistry. 1998; 46(5): 1944-1953.

Chung HY, Yung IKS, Ma WCJ, Kim JS. Analysis of volatile components in frozen and dried scallops (*Patinopecten yessoensis*) by gas chromatography/mass spectrometry. Food Research International. 2002; 35(1): 43-53.

Ciążyńska–Halarewicz K, Kowalska T. A study of the dependence of the Kováts retention index on the temperature of analysis on stationary phases of different polarity. Acta Chromatographica. 2003; 13: 69-80.

Iwaoka W, Hagi Y, Umano K, Shibamoto T. Volatile chemicals identified in fresh and cooked breadfruit. Journal of Agricultural and Food Chemistry. 1994; 42(4): 975-976.

Jin JY, Zhao MY, Yu K, Zhang MT, Wang JM, Hu YT, et al. Squalene acts as a feedback signaling molecule in facilitating bidirectional communication between tea plants. Science Advances. 2025; 11(7): eads4888.

Kanasawud P, Crouzet JC. Mechanism of formation of volatile compounds by thermal degradation of carotenoids in aqueous medium. 1. beta-Carotene degradation. Journal of Agricultural and Food Chemistry. 1990; 38(1): 237-243.

Kawakami M and Kobayashi A. Volatile constituents of green mate and roasted mate. Journal of Agricultural and Food Chemistry. 1991; 39(7): 1275-1279.

Tu NTM, Thanh LX, Une A, Ukeda H, Sawamura M. Volatile constituents of vietnamese pummelo, orange, tangerine and lime peel oils. Flavour and Fragrance Journal. 2002; 17(3).

Vernon F, Suratman JB. The retention index system applied to alkylbenzenes and monosubstituted derivatives. Chromatographia. 1983; 17: 600-604.

Yamaguchi K, Shibamoto T. Volatile constituents of green tea, Gyokuro (*Camellia sinensis* L. var Yabukita). Journal of Agricultural and Food Chemistry. 1981; 29(2): 366-370.

Zhu JC, Chen F, Wang LY, Niu YW, Yu D, Shu C, et al. Comparison of aroma-active volatiles in oolong tea infusions using GC–olfactometry, GC–FPD, and GC–MS. Journal of Agricultural and Food Chemistry. 2015; 63(34): 7499-7510.
